# Enhanced Sensitivity and Resolution in Biomolecular CEST NMR Experiments Using the Extended Hadamard Encoding Scheme

**DOI:** 10.1021/acs.analchem.5c04282

**Published:** 2025-12-17

**Authors:** Jihyun Kim, Micael Silva, E̅riks Kupče, Sundaresan Jayanthi, Adonis Lupulescu, Rina Rosenzweig, Lucio Frydman

**Affiliations:** † Departments of Chemical and Biological Physics, 34976 Weizmann Institute of Science, Rehovot 7610001, Israel; ‡ Department of Chemistry Education, 34986 Kyungpook National University, Daegu 41566, South Korea; § Departments of Chemical and Structural Biology, 34976 Weizmann Institute of Science, Rehovot 7610001, Israel; ∥ 187007 Latvian Academy of Sciences, Alademijas Laukums 1, Riga 1050, Latvia; ⊥ Department of Physics, 195697 Indian Institute of Space Science and Technology, Thiruvananthapuram 695 547, Kerala, India

## Abstract

By untangling information using deterministic frequency-domain linear combinations, the Hadamard Transform (HT) offers a robust way to extract spectral information. Hadamard-based encoding schemes can shorten the acquisition times of nuclear magnetic resonance (NMR) experiments, and lead to substantial sensitivity gains/unit time. However, the presence of spin–spin couplings, spin–spin cross-relaxation, or other mechanisms that break simple one-to-one relationships between a peak intensity and its frequency-domain position can complicate this approach. A case where manipulations at a given peak frequency position may affect the intensity of other peaks in the spectra arises in chemical exchange. Thus, in frequency-domain NMR experiments such as chemical exchange saturation transfer (CEST), saturation at one frequency position may bring about significant intensity changes at another frequency. This study shows that when based on classical frequency-domain Hadamard encodings, strong artifacts will then arise in NMR CEST experiments. The origin of these artifacts is explained and a way to eliminate them while preserving HT’s desirable characteristics is proposed, utilizing an extended HT (eHT) scheme. CEST NMR experiments performed and processed using the eHT are shown to be free from artifacts, while leveraging Hadamard’s sensitivity-enhanced performance over step-by-step frequency-domain implementations. Good performance is also observed when eHT CEST is compared against other accelerated versions of protein CEST NMR. A summary of the features and potential opened by these new experiments is provided.

## Graphical abstract

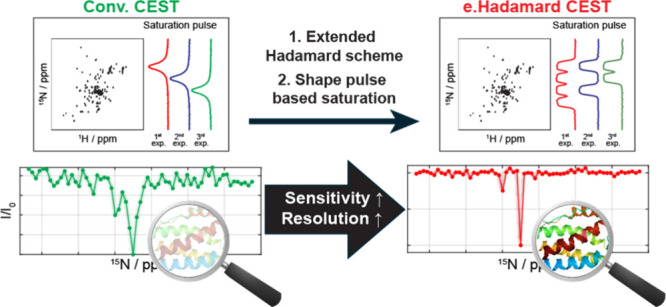

## Introduction

Chemical Exchange Saturation Transfer (CEST) experiments are widely used in both nuclear magnetic resonance spectroscopy (NMR)^1–3^ and imaging (MRI).^4–8^ In both cases they amplify the response of a given minority state, by porting its information in a magnified fashion to a much more abundant reporter. When this information is transferred via chemical exchanges occurring at a rate *k*_ex_, magnification factors on the order of *k*_ex_ *·*T_1_ may arise—T_1_ being the longitudinal relaxation time of the reporting species. This product can amount to enhancement factors of 10–100×, making CEST an important tool to detect low concentration metabolites in vivo, revealing low-populated states in biomolecules,^9–12^ and for materials applications.^13,14^ When targeting biomolecules, CEST is used to detect minor or “invisible” states that are in slow exchange with a major, observable conformation—thus revealing states that are difficult to capture directly using NMR.^9–11^ As in all CEST counterparts, these experiments involve the selective saturation of the minority species at its resonance frequency; this saturation will then transfer via chemical exchange to the magnetization pool belonging to the majority species.^3,4^ By monitoring the resulting decrease in the latter, the presence and spectral characteristics of the low-population states can be measured, and kinetic details about the exchange process can be inferred. CEST experiments targeting various nuclei including ^1^H, ^13^C, and ^15^N have thus been demonstrated, and proven valuable for studying interconversions in proteins, nucleic acids, and other biological macromolecules.^9–12,9–12,15–18^

Despite its magnifying power, biomolecular CEST NMR faces limitations, foremost in terms of experimental time and resolution. This is a consequence of the a priori unknown position of the exchanging partners, meaning that the saturation of the minority sites needs to be probed in a lengthy, step-by-step series of experiments—usually involving the arraying of multiple 2D NMR acquisitions. Achieving accurate CEST profiles also requires that the frequency saturations be relatively weak and closely spaced, making the overall efficiency of the experiment reminiscent of early days high resolution continuous-wave NMR. In an effort to reduce measurement time and improve the efficiency of CEST experiments, DANTE-based multisite excitation was proposed; an experiment that requires a number of repetitions to distinguish the positions of the genuine dips.^19^ On the other hand, the Hadamard Transform (HT) has long been recognized as a powerful approach to improve the efficiency—and thereby the signal-to-noise ratio (SNR) per unit time—of frequency-domain NMR experiments, particularly when targeting indirectly detected dimensions.^20–22^ HT can probe and disentangle signals from multiple frequencies simultaneously, enhancing the SNR thanks to a more efficient use of the experimental acquisition time. This approach has been found particularly efficient for the collection of multidimensional experiments such as COSY, TOCSY, NOESY, HSQC and HNCO, with the Hadamard scheme often allowing one to exploit a priori known 1D NMR information.^23,24^ The utility of this method has also been recently demonstrated in homonuclear multidimensional correlations targeting labile sites in proteins, polysaccharides, and nucleic acids—including studies on SARS-CoV-2-derived RNAs.^25,26^

Given the suitability of the HT scheme to target frequency-domain high-dimensional NMR experiments and given CEST’s reliance on a frequency-by-frequency spectral saturation, this study explores the use of the HT for improving the performance of CEST-based acquisitions. When implemented in its original version, we observe that Hadamard encodings introduce a slew of artifacts in the resulting spectral profiles, which hinder accurate data interpretation. The origin of these artifacts is identified, and experimental refinements capable of overcoming them are proposed. The implementation of the resulting “extended” HT schemes is found to yield clean CEST biomolecular NMR information, while preserving SNR advantages. Results of conventional-, DANTE- and Hadamard-encoded versions of ^15^N CEST were then compared for two proteins – drkN SH3 and hTRF1– undergoing slow-to-intermediate exchanges between folded and unfolded state at different population ratios. In both cases, the extended HT provided good sensitivity, as well as quality kinetic and chemical shift information about the exchanges involved.

## Experimental Methods

### Sample Preparation

The drkN SH3 domain and hTRF1 proteins were overexpressed and purified using protocols as previously described.^27,28^ While the gene for the SH3 domain of *Drosophila melanogaster* enhancer of sevenless 2B protein (drkN SH3) was cloned into the pET-28 vector, the human TRF1 protein (hTRF1), encoding the hTRF1 377–430 fragment, was cloned into the pET-29b plasmid. Both final genes incorporate an N-terminal 6×-His-tag followed by a TEV protease cleavage site.

A culture of BL21­(DE3) cells harboring the drkN SH3 plasmid or hTRF1 was grown at 37 °C in M9 minimal medium supplemented with 1 g/L ^15^N-labeled ammonium chloride and kanamycin (50 mg/L). The culture was grown to OD600 = 0.8–0.9, which expression was induced by addition of 1.0 mM IPTG, to continue overexpression at 25 °C overnight. Bacteria were harvested and the cells were then sonicated under denaturing conditions (i.e., buffer containing 6 M guanidinium chloride). The lysate was cleared by centrifugation and the protein was then purified from the supernatant using a Ni-NTA column equilibrated with the denaturing buffer. The unfolded protein was refolded on the column before elution by lowering the denaturant concentration stepwise from 6 to 4, 2, 1, and 0 M. The His-tag was removed by incubation with TEV protease overnight at 4 °C. Cleaved proteins were separated from the 6×-His-tag and TEV protease by passing over a Ni-NTA column, and further concentrated with an AmiconUltra-15 with 3 K MW cutoff filter (Millipore). Further purification happened on a HiLoad 16/60 Superdex 75 size exclusion column (GE Healthcare) equilibrated with 50 mM Hepes, 300 mM NaCl (pH 7.4). The samples containing protein of interest were pooled, concentrated and flash-frozen. The purity of the proteins was confirmed by SDS/PAGE and conventional 1D ^1^H or 2D ^1^H–^15^N HSQC NMR experiments.

NMR samples of ^15^N-labeled drkN SH3 were freshly prepared at concentrations of 1.2 and 0.1 mM in 50 mM HEPES buffer (pH 7.5) with 50 mM KCl, and 10% D_2_O, 0.03% NaN_3_ and 10 μM DSS. Additionally, a 1.2 mM sample of ^15^N-labeled hTRF1 was prepared in 50 mM HEPES buffer (pH 6.8) with 50 mM KCl, and 10% D_2_O, 0.03% NaN_3_ and 10 μM DSS. For measuring the spatial frequency profiles of the “Hadamardized” pulse, a doped water sample consisting of 1% H_2_O in D_2_O with 0.1 mg of GdCl_3_ and 0.1% DSS was used.

### NMR Spectroscopy

All initial NMR experiments, optimizations, and checkings of the Hadamard pulse were performed on a 498 MHz Magnex magnet interfaced with an 11.7 T Bruker Avance Neo console equipped with a TCI Prodigy probe. All CEST protein NMR experiments, including conventional,^10^ DANTE (D),^19^ and extended Hadamard, were conducted using a 1.0 GHz, 23.5 T Bruker Avance Neo magnet equipped with a TCI cryoprobe. CEST experiments were performed on drkN SH3 at 298.0 K and hTRF1 at 309.5 K. The temperature was previously calibrated using a 99.8% methanol-d_4_ Bruker NMR standard reference sample. A series of 2D spectra were recorded with ^15^N offsets ranging over 3000 Hz (∼30 ppm) centered at 118 ppm, with a total of 64 complex points and acquisition times of *t*_1,max_ = 105 ms and *t*_2,max_ = 70 ms for the ^15^N and ^1^H dimensions, respectively. Each FID was averaged over 8 scans for conventional CEST, while averaged over 4 scans for DANTE CEST (D-CEST) and extended Hadamard CEST experiments. An interscan delay of 1.5 s was used. In the conventional ^15^N CEST experiments, the CEST dimension consisted of 64 points (or 102 points), including the reference plane, as a total of 63 ^15^N frequencies were targeted, separated by 50 Hz (or 101 planes separated by 30 Hz). A CW pulse was used to saturate these frequencies during a *T*_EX_ of 500 ms with γ*B*_1_^eff, CEST^ of 30 Hz for the drkN SH3 sample, or a *T*_EX_ of 300 ms with γ*B*_1_^eff, CEST^ of 20 Hz for the hTRF1 sample. Similarly, for the drkN SH3 sample, ^15^N D-CEST data sets were recorded using an effective γ*B*_1_ field $\left(\gamma B_{1}^{D-\mathrm{CEST}}\frac{\tau_{\rho }}{\tau^{’}}\right)$ of 30 Hz during the CEST element (*T*_EX_ = 500 ms); here τ_ρ_ is the DANTE pulse width, applied at a strength γ*B*_1_^D–CEST^ = 6.02 kHz, and τ^’^= 1/sw_CEST_ is the time-delay between DANTE pulses. For the hTRF1 sample, an effective R ${\gamma B}_{1}^{D-CEST}\frac{\tau_{\rho }}{\tau^{’}}$ of 20 Hz was used, *T*_EX_ = 300 ms, and DANTE pulses were applied using a 5.88 kHz field. The ^15^N D-CEST field position was varied over two different sw_CEST_ values (780 and 810 Hz) with a step-size of 30 Hz to generate a pseudo-3D CEST data set containing 57 2D planes in total. CEST-related parameters are summarized in Table S1; notice that these parameters were optimized for the best realization of the individual experiments. Extended Hadamard CEST data was acquired using the pulse sequence shown in Figure 1a, modified from Vallurupalli et al.^10^ to include Hadamard-encoded pulses for saturation. A pair of Hadamard CEST experiments were recorded where a total of 63 ^15^N frequencies were addressed in either an +H64 or inverted H64 (−H64) matrix—leading to a CEST dimension size of 63, the same as in the conventional case. The CEST profiles obtained from the extended Hadamard CEST scheme were obtained by summing the profiles extracted from the two data sets measured using +H64 and −H64. For saturation, a PC9 pulse^29^ with a bandwidth of 40 Hz and a duration of 180 ms was chosen and Hadamard encoded based on the +H64 or −H64 matrix. Alternatively, the same number of ^15^N frequencies can be encoded into two +H32 matrices, with odd and even-ordered frequencies (now ^15^N frequencies are spaced by 100 Hz) separated into different H32 matrices. In this case, a total of 4 experiments were performed (+H32, +H32′, −H32, −H32′). For these, instead of the PC9 pulse, a *sinc450* pulse was chosen to generate the Hadamard saturation pulse with a bandwidth of 28 Hz and a duration of 101 ms. These Hadamard pulses were generated using WaveMaker via the “wvm_x -a” command. A detailed protocol, including pulse sequence and experimental setup, is available on https://www.weizmann.ac.il/chembiophys/Frydman_group/software.

**Figure 1:**
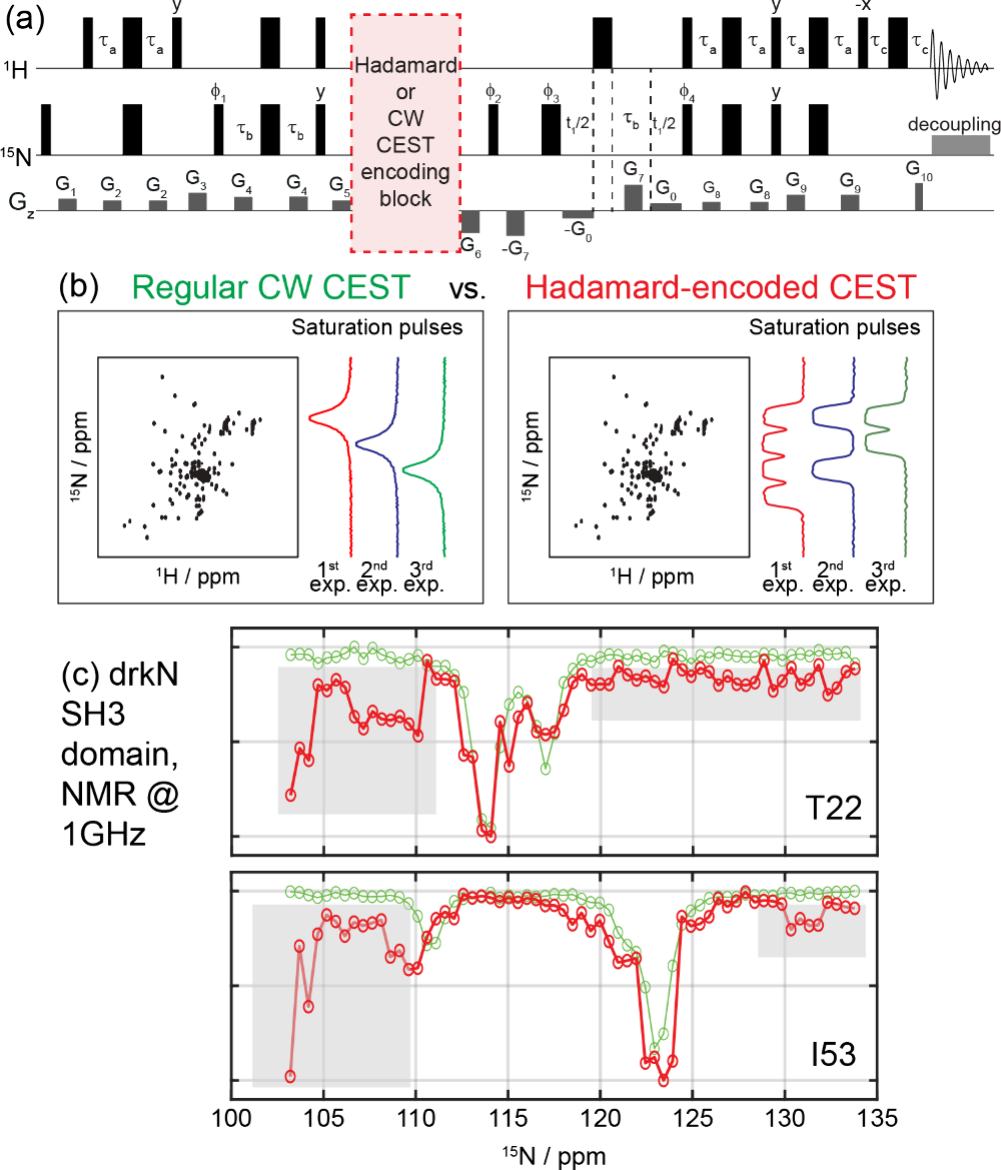
(a) Adaptation of a previously reported pulse sequence^10^ to the aims of this study. During the ^15^N longitudinal storage period (red highlight), either a weak γ*B*_1_ CW pulse targeting a single ^15^N frequency or a polychromatic pulse made up by a sum of weak square or 90°-shaped pulses followed by crushing gradients was used. (b) Schematics illustrating these regular (left) vs Hadamard-encoded (right) CEST experiments. In regular CEST, a series of 2D HSQC spectra is acquired with frequency-specific ^15^N RF saturation pulses (indicated as 1^st^, 2^nd^, and 3^rd^ experiments) that are stepped throughout the spectrum. In Hadamard-encoded CEST, the acquisition is performed in the same number of scans, but each scan uses a polychromatic pulse where half of all ^15^N frequencies is encoded (saturated). This should provide a sensitivity enhancement on the order of √(#bins)/2. (c) Comparison of regular (green) and Hadamard-encoded (red) CEST profiles measured for two representative residues, on a 1.2 mM drkN SH3 protein sample using a 1.0 GHz NMR at 298 K. In both experiments, an effective ^15^N γ*B*_1_ saturation field of 30 Hz was applied during 500 ms at each frequency bin, with a total of 63 frequency elements (points) separated by 50 Hz targeted in the CEST dimension. FIDs for each t_1_ increment were averaged over 8 scans. Unlike the traditional CEST profiles, patterns reconstructed after applying an HT on the polychromatic CEST acquisitions show a series of background artifacts and noises (highlighted in gray (superimposed on the legitimate main (self) and minor (exchange) dips)). Similar Hadamard-derived artifacts were observed for other residues; see the Experimental Methods section for further details.

## Results and Discussion

### Potential and Problems Associated with HT CEST NMR

Figure 1 compares the idea underlying Hadamard-encoded CEST vs a traditional CEST acquisition. For simplicity, we assume a common scenario whereby the CEST experiment seeks to identify minor states in a protein by targeting its ^15^N NMR frequency domain, in an experiment where these intensity changes are detected using ^15^N–^1^H 2D NMR. Instead of saturating each ^15^N frequency one-by-one, the HT scheme will rely on polychromatic saturation pulses where all targeted frequencies are encoded as either present (not saturated) or absent (saturated) in every scan, according to the zeroes and ones in a Hadamard matrix of proper dimensionality (Figure 1b). The effective individual saturation fields and the number of binned frequencies in this matrix will be assumed set as in the conventional CEST NMR; however, as on average half of all frequencies are saturated in every scan, the HT should provide a substantial sensitivity enhancement over an experiment that devotes each scan to probe solely one frequency. Then, an HT reconstruction of the data should lead to a spectral array corresponding to measurements at each ^15^N saturation frequency that was binned. Aside from an SNR improvement, this should be the same kind of 2D spectral array as the one that would arise using conventional CEST.

To explore this possibility, the profiles from traditionally- and Hadamard-encoded CEST experiments were recorded on the SH3 N-terminal domain of the Drosophila protein Drk (DrkN SH3). This is a marginally stable (Δ*G*(*U*) = 1 kcal/mol) protein that at 298 K exists in equilibrium between folded and unfolded states, undergoing slow exchanges on the NMR chemical shift time scale (*k*_ex_ ≈ 2.2 s^–1^).^30,31^ Conventional and HT CEST experiments were measured at the same temperatures, using equivalent saturation radiofrequency (RF) fields (γ*B*_1_/2π = 30 Hz) achieved in one case by a single continuous-wave (CW) pulse, and in the other by a sum of polychromatic pulses whose nonzero components were updated from increment to increment (Figure 1b). In addition, both schemes used the same number of frequency bins (“points”) separated by identical increments along the CEST dimension, and the same overall scanning times. This sample was relatively concentrated and was detected at 1.0 GHz NMR using a 2D ^15^N/^1^H heteronuclear correlation experiment, based on the refocused INEPT sequence described in ref 31. (Figure 1a). Thanks to the good ensuing sensitivity, emphasis could be placed on facilitating the clear, unambiguous identification of major and minor states as in the “dips” observed in the CEST traces.

Figure 1c compares these profiles as measured using the CW and HT saturation approaches; clearly, the latter not only fails to improve SNR, but shows many artifacts and noises that should not be there. Overall, we identified three primary sources of artifacts in these Hadamard CEST experiments. These are (1) overlap and crosstalk among the saturation pulses; (2) noises associated with the use of square CW RF shapes to construct the polychromatic Hadamard waveform; and (3) crosstalks among multiple exchanging sites leading to artificial CEST-like peaks and noises. The nature of these artifacts and strategies to mitigate them are discussed below.

### Encoding a Continuous Frequency Axis via “Hadamardized” Saturation/Excitation Pulses: Crosstalks within the Polychromatic Pulse

To identify the sources that contribute to the artifacts and noises in Figure 1c, it is enlightening to examine the frequency selectivity that will arise when trying to measure a spectrum via “Hadamardized” saturation pulses. This is somewhat different from Hadamard encodings used in the past for encoding NMR information, where the targeted sites possessed a priori known frequency positions:^25,26^ in the present case, the resonance frequencies of the various sites are unknown, and hence a series of regularly spaced, contiguous frequency elements needs to be used—and then disentangled. It is illustrative to explore how well Hadamard-encoded polychromatic pulses will extract such densely packed 1D information. A simple way of measuring this is by “imaging” a water sample, which is subject to a 1D gradient for a long enough time to perform a suitable saturation on it. When this is done by polychromatic pulses whose frequency components have been Hadamard-encoded, a suitable Hadamard-based reconstruction should provide—when imaged—a resolved representation of the single-frequency targeted components. Figure S1 (Supporting Information) shows the pulse sequence used in such an experiment, and the single-frequency saturation “peaks” that the ensuing slices yield after Hadamard reconstruction. Notice in Figure S1c the strong baseline artifacts observed when saturation fields that are closely spaced (γ*B*_1_/2π = 30 Hz, interfrequency separation = 50 Hz) are used to compose the polychromatic pulses; these artifacts are responsible for part of the SNR losses evidenced in Figure 1c, and only become negligible when RF fields are much smaller than the interbin separation (e.g., γ*B*_1_/2π = 5 Hz).

Supporting Figure S2 revisits in more detail the nature of these strength-dependent RF artifacts, for a simple scenario composed of four frequencies to be unraveled by saturation—once again using the simple gradient-based readout. When the polychromatic saturation combines relatively strong RF pulses (γ*B*_1_/2π = 20 Hz, Figure S2a), each frequency bin is well covered, but there is significant overlap between the “tails” of the saturation pulses; this leads to artifacts after their HT. By contrast, use of weak RF fields to build the polychromatic pulses (e.g., γ*B*_1_/2π = 5 Hz) leads to minimal crosstalks and no artifacts after the HT; however, in such cases, the saturation pulses provide an incomplete saturation of each frequency bin. This also leaves some frequency regions barely addressed, raising the possibility that genuine peaks are missed if sited in-between saturation frequencies (e.g., the regions marked in gray in Figure S2b).

It follows from this that upon combining strong or weak RF saturation pulses in a Hadamard fashion, there may either be “leakage” between the bins leading to imperfect cancelations and noise, or the possibility that genuine signals may be lost between the applied frequencies. In order to achieve a more optimal “square-shaped” binning, we explored the possibility to “crush” the binned signals using a variety of shaped pulses—including sinc,^32^ E-BURP,^33^ and PC9-shaped pulses. Figure 2 compares how a choice based on PC9 excitation pulses followed by crusher gradients performs against CW-based saturation pulses—using again the imaging of a four-bands encoding as example. In both cases ca. 40 Hz wide bins spaced 50 Hz apart were targeted, using Hadamard encoding blocks lasting ca. 500 ms. As can be seen by comparing Figure 2b,d, the separation of the bands is much clearer in the former case than in the latter. As a side benefit, approaches based on selective 90° + crusher gradient pulses, ended up requiring less power than CW-based Hadamard pulses. For example, when applying a CW-based Hadamard pulse encoding based on 32 individual 30 Hz nutation fields to construct the H64 matrices, the γ*B*_1,max_ ended up being 1890 Hz (over 9 W on our system). By contrast, building similar matrices based on Hadamardized PC9 excitation pulses only required 696 Hz fields, depositing 1.5 W of power. This reduction eases greatly the operational demands, particularly when applying this method at high fields (≥1 GHz), where wider frequency ranges (i.e., more frequency components) need to be covered and where dielectric losses are more severe.

**Figure 2:**
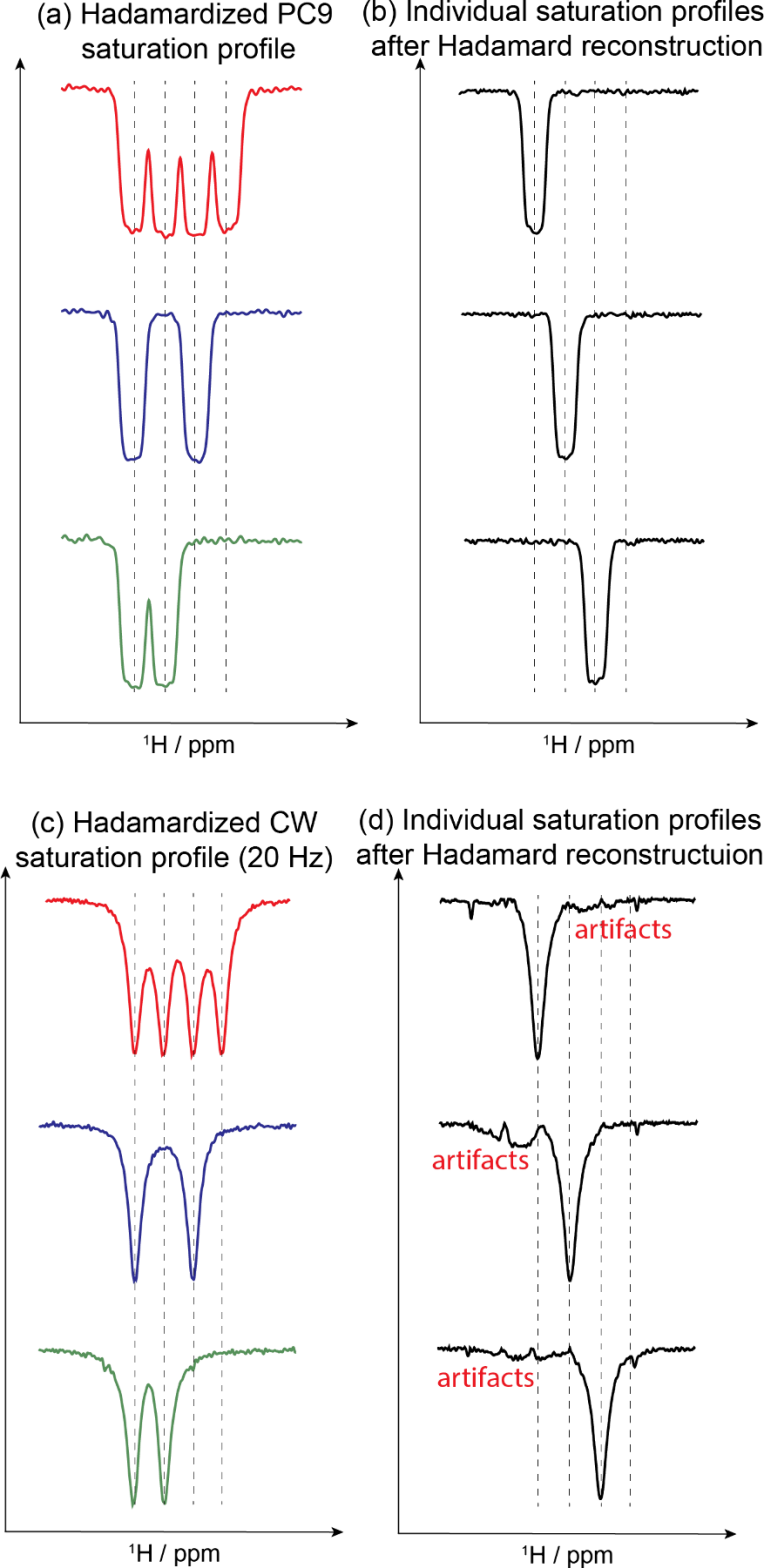
Saturation profiles measured for a four-component separation using Hadamardized schemes based on PC9+crusher 90° pulses (a,b), vs CW saturation (c,d) pulses. The pulse sequence used in this water-based experiments is as in Figure S1a, with either weak γ*B*_1_ square pulses targeting the predefined frequencies or a PC9-based Hadamardized 90° pulse, repeated three times for ensuring good “saturation.” In the PC9 case, this was done by three 180 ms long 90° pulses plus gradient crushers, and in the CW by 500 ms long pulses. (b,d) Hole-burned profiles of each individual frequency after Hadamard processing; notice the different levels of artifacts.

### Second Source of Artifacts: Crosstalks among the Exchanging Sites

Even with the above precautions, artifacts will still arise in the final HT-derived CEST profiles. This is illustrated in Figure 3, which compares selected 1D profiles arising from 2D ^1^H–^15^N CEST NMR collected with conventional and with Hadamard-encoded means on the drkN SH3 protein. The provisions of the preceding section did indeed remove the strong baseline noises that arose before in the reconstructed 1D profiles, and both the self- and the exchange-transferred saturation peaks are clearly visible in the HT experiment. Also visible, however, are minor peaks that should not be present, and which could lead to a false identification of the exchanging states. The presence of these additional artifacts can be traced to yet another kind of crosstalk—this one arising from interferences among multiple exchanging sites. Indeed, a central assumption underlying HT NMR is that addressing a particular frequency by either excitation, inversion or saturation, will only influence the response associated with that spectral component: no other frequency response should change because of the presence or absence of such manipulation. However, if crosstalks exist among the peaks at independent frequencies—for instance, if saturating the frequency of an “invisible” site changes the response of a “majority” site positioned elsewhere– this assumption will be broken; and so will be the accuracy of the HT reconstruction. This phenomenon was recently observed in NOE experiments when applying polychromatic saturation pulses on sites that are mutually cross-relaxing;^26^ the same phenomenon will arise in CEST—not due to cross-relaxation, but rather due to cross-saturation between exchanging sites.

**Figure 3:**
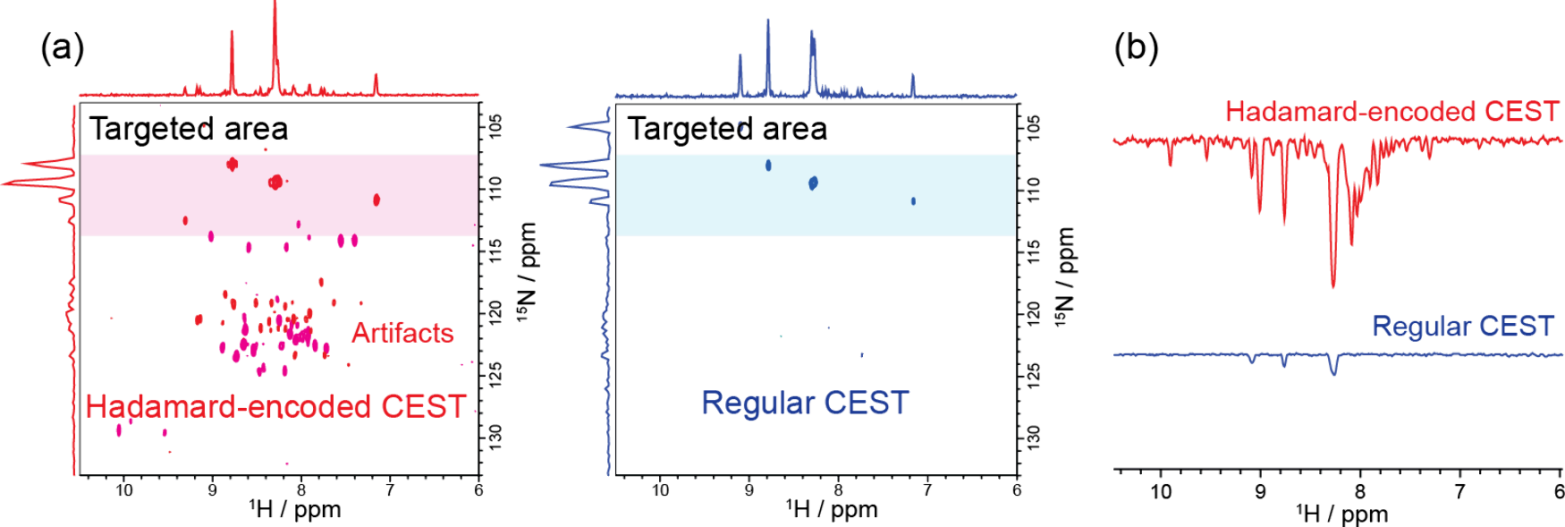
(a) 2D planes extracted from Hadamard- (left, in red) and conventionally encoded (right, in blue) CEST experiments acquired on 1.2 mM drkN SH3 protein at 298 K using a 1.0 GHz NMR spectrometer. The selected 2D planes correspond to measurements where RF irradiation was targeted around the shaded ∼110 ppm region, with γ*B*_1_^eff^/2π of 15 and 30 Hz, respectively. To enhance the visibility of the saturated peaks, the phase of the 2D spectra were inverted in this figure; shown on the sides of the 2D plots are projections of the individual CEST sets along the two axes. (b) 1D projections extracted from all spectral regions of the 2D spectra shown in (a), highlighting the presence of artifacts with the Hadamard encoding.

It is possible to remove these artifacts using an extended version of the Hadamard matrix, whereby the results of two Hadamard-encoded nxn matrices, which we denote as +Hn and −Hn, are suitably coadded. These matrices would correspond to two HT CEST experiments that are identical in every way, except for the fact that one is the logically negated version of the other—i.e., with all saturated and nonsaturated elements inverted in the two coadded parts of the overall acquisition. When implemented on noninteracting sites such experiments would provide redundant information: there is no information in the negated version of a Hadamard encoding matrix, that is not present in the original Hadamard matrix. But it has been shown that when frequency bins interact with one another, this negation can help cancel the multisite crosstalks, while reinforcing the genuine signals.^26^ Figure 4 illustrates the effectiveness of this “extended HT scheme,” focusing once again on the drkN SH3 domain. CEST experiments encoded with either the +H64 or −H64 Hadamard matrices incorporating opposite saturation profiles give identical behaviors for the genuinely exchanging sites. They also originate artificial resonances (Figure 4a, circled features), but the signs of these artifacts are opposite in the +H64 and −H64 experiments. Crosstalk artifacts are thus readily eliminated by summing these two profiles, while the genuine features coherently add up. The cleanness of the ensuing extended E64 = [(+H64) + (−H64)] HT combination is visible in Figure 4b, which shows the postacquisition cancelation of the artifacts, leaving only the genuine peaks in the final traces. Supplementary Figure S3 presents a complementary analysis, similar in all its aspects to Figure 4, but upon using CW-based Hadamardized pulses for the saturation. As with the PC9-based case shown in Figure 4, artifacts here also arise with opposite signs when measured using the traditionally encoded Hadamard scheme; but they also cancel out well in the extended HT CEST profiles. Notice however that, despite this improvement, the resulting profiles still show noisier backgrounds than the PC9-based case shown in Figure 4; this reflects the aforementioned crosstalk within the polychromatic CW-based saturation pulses.

**Figure 4:**
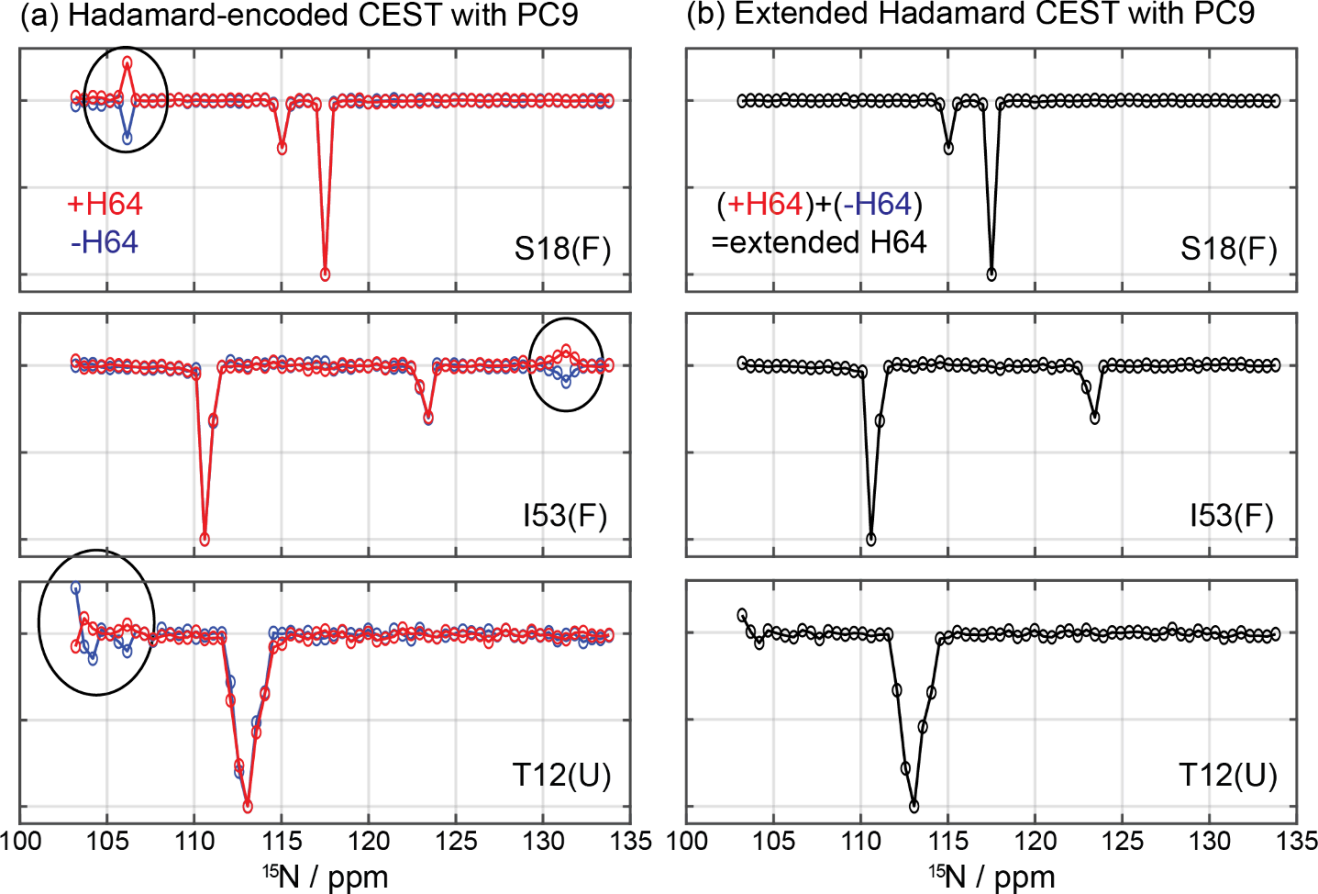
(a) CEST profiles of the drkN SH3 protein measured using the Hadamard CEST pulse sequence shown in Figure 1a. Profiles shown in red were obtained using the regular H64 matrix (+H64), while profiles shown in blue were obtained using the negated H64 matrix (−H64)—where all bins that were saturated in +H64 are left untouched and the ones that were untouched are now saturated. Upon subject these data to a Hadamard decoding, the profiles measured with +H64 and −H64 exhibit opposite phases between artifacts (black circles) but identical responses for the genuine signals. (b) CEST profiles arising from such extended Hadamard acquisition, obtained by summing the +H64 and −H64 profiles. Note the removal of the artifacts; see text for additional details.

### Sensitivity and Line Shape Considerations

Enhancing sensitivity per unit time was the main driver of this work. Figure 5 compares the sensitivity performances of the extended Hadamard CEST against a regular CEST acquisition, using once again the drkN SH3 protein as a test example. For the sake of completeness, the comparison includes profiles obtained using D-CEST experiments, which use a multipulse irradiation and unfolding scheme to significantly reduce the required experimental duration—hence also increasing the effective signal-to-noise ratio (SNR) per unit time.^19^ The regular CEST experiment was performed using a weak CW irradiation field of 30 Hz and the D-CEST was run using the excitation scheme with a DANTE element, comprising high power short pulses separated by delay τ′ $\left(\gamma B_{1}^{D-\mathrm{CEST}}\frac{\tau_{\rho }}{\tau^{\prime }}=30\;\mathrm{Hz}\right)$ . Although these were nominally narrower than the bandwidths of the 90° PC9 pulses used in the extended Hadamard encoding scheme (40 Hz), the latter’s sharper edges bring about a resolution improvement in the final traces. When performed on the more concentrated, 1.2 mM protein sample, the conventional and Hadamard-derived profiles reveal both folded and unfolded states at similar positions, without any additional artifacts. However, the relatively concentrated nature of this sample makes an accurate assessment of the SNR merits for the various experiments difficult. To better assess these, 1.0 GHz measurements were repeated under identical conditions on a 0.1 mM sample (Figure 5b). Under these conditions, the advantages of the new scheme in terms of both sensitivity and resolution, become clearer. Complete comparisons of the profiles from the three different acquisition schemes, for both high and low concentrations of this protein, are shown in the Supporting Information for every cross peak resolved in the 2D ^15^N–^1^H correlation (Figures S4 and S5, respectively). Notably, the extended HT CEST profiles of all residues exhibit similar improvements in sensitivity and resolution as the one shown in Figure 5, confirming the robustness and broad applicability of the new method across the entire protein.

**Figure 5:**
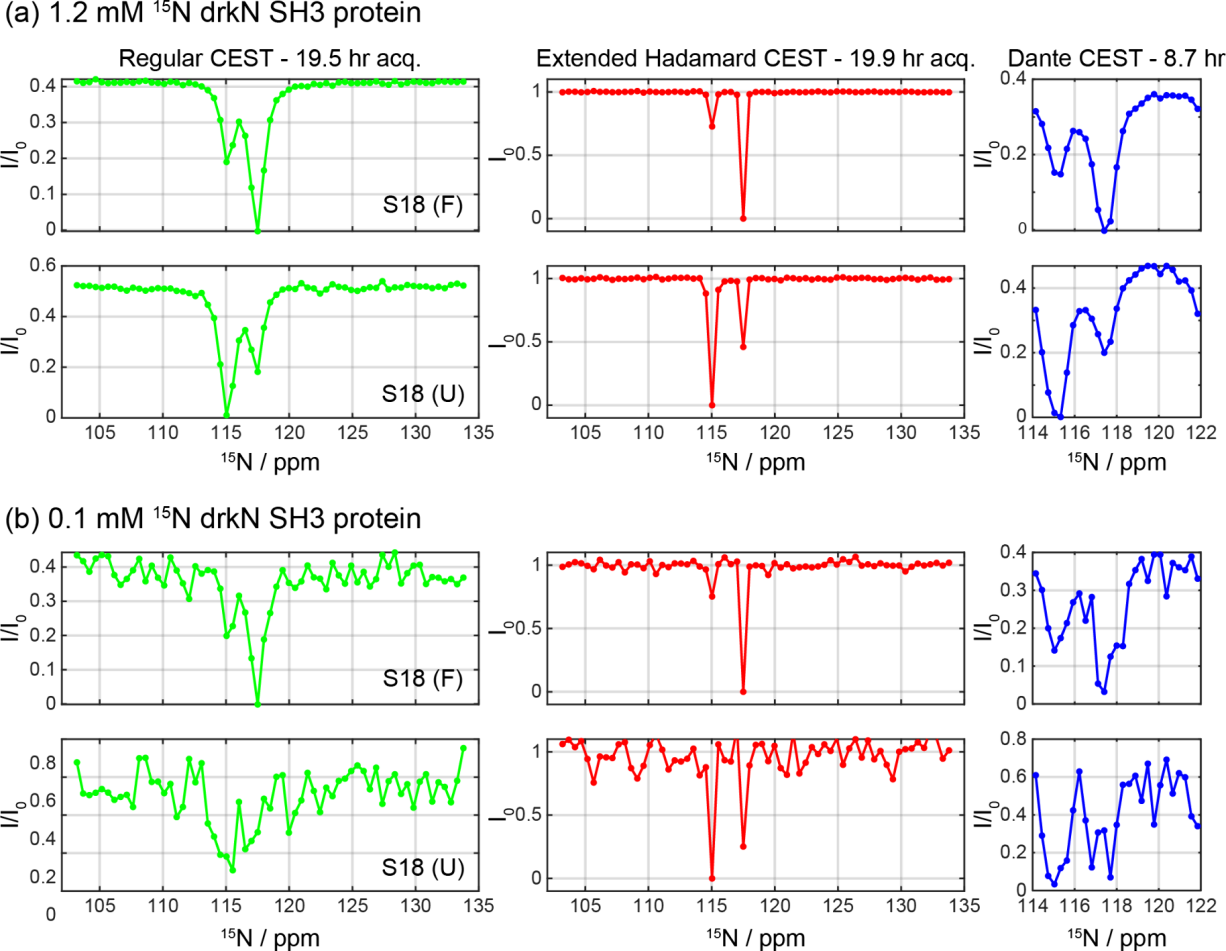
^15^N CEST profiles of residue S18 in both the folded and unfolded states of the drkN SH3 protein at concentrations of (a) 1.2 mM and (b) 0.1 mM. All data were recorded at 298 K on a 1.0 GHz NMR. For D-CEST, only one data set is displayed here but actually two with different spectral widths were acquired, for a total of ca. 18 h. Profiles for both samples and for all residues resolved in the protein are presented in the Supporting Information.

These experiments indicate that the extended HT endows CEST with SNR improvements; ideally, the enhancements should be on the order of √(#bins)/2, but the actual performance may be degraded by noises associated with inefficient saturation and crosstalk effects. To assess these, SNR calculations of the CEST profiles were conducted as illustrated in Figure S6: the profiles from both conventional and extended Hadamard CEST were plotted in absolute intensity, with the intensity dip of the major state set to the maximum value. SNR was then calculated for the ensuing profile as maximum_peak_intensity/σ_noise_, and SNR per unit time was calculated as SNRT = SNR/√(total experimental time). Residue-specific SNR and SNRT comparisons between the conventional and extended Hadamard CEST spectra emerging from this procedure for both high and low concentration samples of drkN SH3, are shown in Figure S7. For most peaks, the new scheme demonstrates superior SNRT, with overall Hadamard improvements ranging between 2.3 and 2.6 times compared to the conventional counterpart, for the high and low concentration samples, respectively. In either case this gain is less than the factor of 4 expected solely based on the size of the (H64) Hadamard matrix; we ascribe these to the aforementioned pulse-related factors. Remarkably, there is a heterogeneity in the SNR/SNRT gains – which range from ca. 10x for some residues, to instances where the regular CEST scheme performed better SNR-wise. We ascribe this to kinetic details of both the F ⇌ U exchange and of the interchanges of the labile residues with the solvent, which may make saturation transfers based on a CW pulse irradiation different from those associated with a train of 90°-crusher pulses (vide infra). Notice that these calculations did not include the D-CEST profiles in the comparisons, as the CEST spectral widths arising from these experiments are very different from the other profiles.

### hTRF1—Protein with Very Different Exchanging Populations

In the case of SH3, the populations of the folded and unfolded states are significantly high and in slow exchange, to the point that their peaks can even be detected on conventional 2D experiments. To further assess the performance of the extended HT in a case where the minority-state population is “invisible” unless highlighted by CEST, experiments were repeated on hTRF1—a protein that at *T* = 308 K has a minority unfolded population of approximately 4% and a faster exchange (*k*_ex_ ∼ 250 s^–1^).^27^ Due to these conditions, the intensity dip corresponding to the minor state is expected to be much smaller than in the SH3 case. While we anticipated that the extended Hadamard CEST would help identify these weak minor dips thanks to an improved sensitivity, the new scheme failed to detect this minority population when using the PC9-shaped polychromatic pulses. By contrast, conventional CEST revealed these peaks without problems (Figure 6). This difference between the conventional and Hadamard CEST encoding was traced to the small effective γ*B*_1_ fields that the PC9 pulses impart at any given frequency. For the example in Figure 6, the 40 Hz PC9 bandwidth used led to a γ*B*_1_^eff^ of only 2.2 Hz—too weak to generate significant changes in the main resonance for these low-population states.

**Figure 6:**
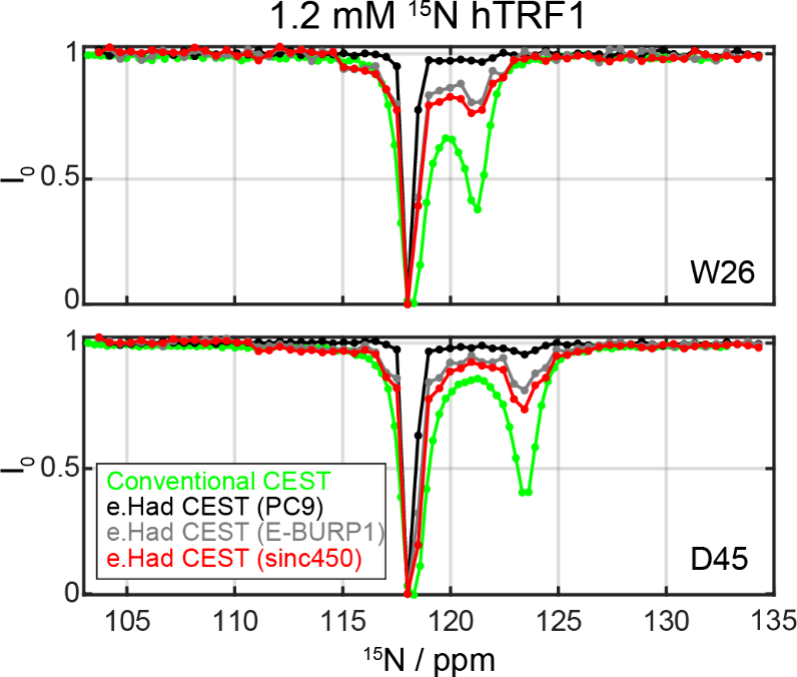
Comparison of ^15^N CEST profiles measured on a 1.2 mM hTRF1 protein sample using different shaped pulse-based extended Hadamard CEST: black: PC9 (BW = 40 Hz, 180 ms, γ*B*_1_^eff^/2π = 2.2 Hz), gray: E-BURP1 (BW = 25 Hz, 180 ms, γ*B*_1_^eff^/2π = 6.3 Hz), and red: *sinc450* (BW = 25 Hz, 113 ms, γ*B*_1_^eff^/2π = 19 Hz). The profile measured using conventional CEST with γ*B*_1_/2π of 20 Hz and a duration of 300 ms is shown in green as a reference. All measurements were conducted at 309.5 K on a 1.0 GHz NMR spectrometer.

From the perspective of its saturation capabilities, nothing will perform better per unit irradiation time than a conventional, CW-based square pulse. Still, even when relying on the pulse/crushing scheme, the effective saturation imparted by the Hadamardized encoding can be improved by changing the type and shape of the pulse. Using an E-BURP1 pulse with a bandwidth of 25 Hz for instance, leads to an effective γ*B*_1_^eff^ of 6.5 Hz (as calculated by computing the time-averaged γ*B*_1_), while using *sinc450* with the same bandwidth achieved a γ*B*_1_^eff^ of about 19 Hz. As the effective γ*B*_1_ increases the intensity dip caused upon hitting the minority state also increases, becoming more prominent in the ensuing spectra (Figure 6).

The effective γ*B*_1_ of the shaped pulses can be increased further by enlarging their excitation bandwidths, opening another way to improve saturation in these experiments. Enlarging the excitation bandwidth, however, would bring back the problem of crosstalks within the polychromatic components involved in the Hadamard encoding; to avoid this, increasing bandwidths would have to be associated with spreading out the spacing between the binned frequencies, thereby sacrificing the superior resolution shown by the Hadamard-encoded CEST traces. One possible way to avoid this, and achieve stronger saturation while preserving high spectral resolution, is to split—at the expense of SNRT—a single Hadamard experiment into two or more interleaved encodings. For instance, selecting either even or odd frequency bins in an H64 encoded experiment, will lead to two H32 and H32′ experiments where the H64 odd- and even-ordered frequencies are encoded separately, and thereby the neighboring frequency spacing could be doubled compared to H64. Figure S8 illustrates the ensuing procedure, once again on experiments that image a water sample under the action of a gradient. An original H64 experiment based on *sinc450* pulses spaced 50 Hz apart with effective fields of 19 Hz, was split here into two H32 experiments where frequency spacing is increased to 100 Hz, and an increased γ*B*_1_^eff^ = 21 Hz. The gains in saturation efficiency coupled to the decrease in artifacts in this latter case, are evident. Such an effective field is also sufficient to target low-populated minor states, such as those in the hTRF1 protein. Figure S9 shows the profiles resolved in this manner for all residues of hTRF1, measured using two extended Hadamard (±H32, ±H32′) CEST experiments in this way. All the minor states observable in the conventional counterparts are also clearly visible in the final profiles, with an improved resolution. The SNR as measured for the major dips in these CEST traces is also ca. 2× higher for these Hadamard-encoded experiments than for conventional CEST experiments recorded in similar acquisition times (Figure S10).

### Extracting Exchange Parameters from Hadamard-encoded CEST NMR

CEST NMR is not only utilized to highlight minority states: interpretation of its line shapes provides insight into the kinetics and thermodynamics of exchange processes, as well as the shifts of the interchanging forms.^10,16,18,34^ This requires knowledge of ancillary parameters such as relaxation times and the number of exchanging sites, but much of the remaining analysis is deterministic. The same should happen in the Hadamard-encoded CEST experiments, where kinetic, thermodynamic, and chemical shift parameters should be amenable from the data given the full details of the experiment, based on Bloch-McConnell exchange model simulations.^35,36^ To confirm that this is the case, data obtained for the drkN SH3 and hTRF1 proteins were quantitatively analyzed—in both cases starting from the conventional and DANTE CEST results, which were used as “ground truths”. For the case of drkN SH3 a fixed unfolded population *p*_U_ = 0.50 ± 0.05 was taken based on the intensities observed in the ^15^N,^1^H HSQC spectrum; fitting both the conventional and D-CEST profiles by ChemEx (https://github.com/gbouvignies/chemex) resulted then in very good agreement for both the exchange parameters (*k*_ex_ = 3.69 ± 0.08 and 3.32 ± 0.08 s^–1^, respectively) and ^15^N chemical shift value differences (Δω; Table S2 and Figure S11). These numbers were also consistent with previous reports.^28,31^ For the case of hTRF1 *p*_U_ was not fixed but rather fitted with *k*_ex_ for the residues with the clearest CEST profiles, and then fixed to fit local Δω for all remaining residues. The ensuing fitting of both conventional and D-CEST on a per residue basis, resulted in consistent exchange parameters: *k*_ex_ = 275 ± 5 and 268 ± 5 s^–1^, respectively, and *p*_U_ = 0.0727 ± 0.0004 and 0.0764 ± 0.0004, respectively. The Δω obtained from both experiments were also consistent (Table S3 and Figure S12), and agreed with previously reported values.^27^

Not surprisingly, we found that when performing a similar analysis for the extended Hadamard CEST data, the a priori known details of the polychromatic selective pulses also had to be included in the simulations. Moreover, to accurately interpret the results, tracking the evolution of the exchanging magnetizations during the cascades of polychromatic pulses used throughout the encoding, and subjecting the ensuing responses to the same HT as done on the experimental data, was also needed. With these provisions, excellent agreement could be obtained between simulated CEST profiles and experimental ones—leading in fact to refinements over conventional approaches. Figure 7 illustrates this for some representative residues in drkN SH3. This Figure focuses on profiles arising upon targeting resonances corresponding to the folded species in the 2D ^15^N/^1^H correlation, and compares the experimental observations (in black) with simulated outcomes (in red) emerging from two sets of assumed parameters: the left-hand column was derived using parameters determined by analysis of the conventional CEST experiments, while the right-hand column simulations were achieved by slightly altering (<0.1 ppm changes) in the ^15^N chemical shifts of the folded and/or unfolded states. The improvements in the latter case are noticeable, confirming that the superior resolution and good sensitivity of the Hadamard-encoded CEST profiles can be exploited to improve the accuracy of the chemical shift predictions.

**Figure 7:**
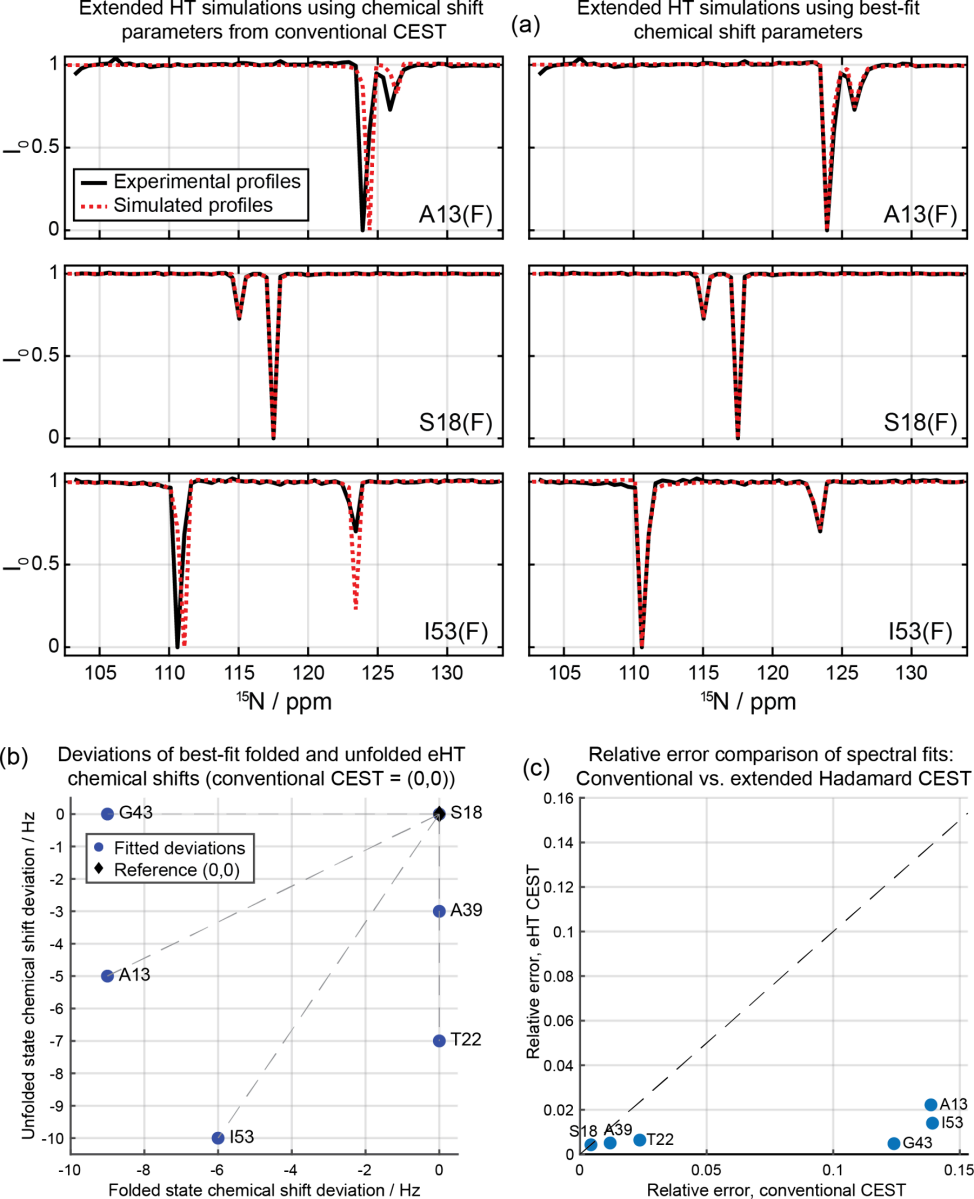
(a) Comparison of ^15^N experimental (black) and simulated (red) Hadamard CEST profiles for selected residues in drkN SH3. The left-hand column plots simulated profiles computed with chemical shifts obtained with conventional CEST experiments; the right-hand column plots simulated profiles computed with slightly modified chemical shifts. Experiments and simulations employed PC9 pulses with (γ*B*_1_^eff^)/2π = 2.2 Hz and 180 ms duration. The frequency changes in Hz (ppm) for major and minor conformations are A13 {−9 (0.09), −5 (0.05)}, S18 {0(0), 0(0)}, I53 {−6 (0.06) −10 (0.1)}. (b) Chemical shift deviations imparted on the folded and unfolded chemical shift values of selected residues by conventional CEST, in order to obtain a perfect match with the Hadamard-encoded CEST experiments. Each vector originates from the conventional CEST reference position (0, 0) and points to the fitted chemical shift deviations for the folded (*x*-axis) and unfolded (*y*-axis) species. (c) Correlation plot comparing the fitting errors of conventional and extended Hadamard CEST experiments, when calculated as $\mathrm{error}=\sqrt{\frac{\sum (E(k)-S(k){)}^{2}}{N}}$ . Here, *E*(*k*) and *S*(*k*) represent the experimental and simulated CEST profiles at frequency point *k*, and *N* is the total number of frequency points. Note that all data points fall below the *y* = *x* line confirms the consistent reduction in error achieved by the Hadamard CEST scheme.

## Conclusions

By bringing the benefits of Hadamard encoding to frequency-domain CEST experiments, this study sought to enhance overall sensitivity per unit time, and in the process improve the information conveyed by CEST profiles. By multiplexing the saturation of multiple frequencies, the Hadamard scheme imparted on CEST a gain in the sensitivity—provided that artifacts were suitably dealt with. Two primary sources of artifact were identified and addressed. This involved replacing the CW square pulses—which are very efficient in terms of saturation but have strong crosstalks—with shaped pulses. A variety of 90°/crusher combinations could then be chosen and tailored to the rate of the chemical exchange, although this would eventually also encounter crosstalk limitations. A simple way to address the latter was by splitting the Hadamard encoding into multiple experiments; this permitted use of higher γ*B*_1_^eff^ values, excellent site resolution and a controllable degree of saturation. A second, more fundamental source of artifacts required extending the HT scheme by combining +Hn with −Hn acquisitions. The ensuing extended HT procedure cleaned the remaining artifacts, while also offering ample opportunities for tailoring the shape, number and effective bandwidth of the Hadamard-encoded pulses. The implementation and processing of this experiment can be downloaded from https://www.weizmann.ac.il/chembiophys/Frydman_group/software.

Being entirely deterministic, simulations could accurately replicate the experimental profiles obtained from these extended Hadamard CEST schemes. When applied to the investigated systems these reproduced with high accuracy the experimental lineshapes. These simulation scripts are available from us upon request; for enabling routine applications, it is likely that an efficient automatic procedure to extract chemical shift and exchange parameters using these scripts should be added to these calculations. We would be happy to collaborate with interested parties in this endeavor.

## Supplementary Material



## Notes

Experimental parameters used for all CEST experiments; saturation profiles of non- and Hadamardized pulses; CEST profiles measured by conventional, DANTE, and extended Hadamard CEST for drkN SH3 and hTRF1 proteins; illustration of SNR calculations for the CEST profiles; comparison of SNR between conventional and extended Hadamard CEST profiles; and ^15^N chemical shift differences (Δω) between the F and U states and their correlation plots for drkN SH3 and hTRF1 proteins (PDF)

## #

Department of Pharmaceutical Sciences, University of Vienna, Josef- Holaubek-Platz 2, 1090 Vienna Austria
