# Supplementary material for: Enhanced Sensitivity and Resolution in Biomolecular CEST NMR Experiments Using the Extended Hadamard Encoding Scheme
Source: Anal Chem. 2025 Dec 17;97(51):28192–202. doi: 10.1021/acs.analchem.5c04282 (PMC12756851; doi:10.1021/acs.analchem.5c04282)
Supplement: Supplementary file 1 [file ac5c04282_si_001.pdf]

Supporting Information for

Enhanced Sensitivity and Resolution in Biomolecular CEST NMR Experiments Using the Extended Hadamard Encoding Scheme

Jihyun Kim[1,2], Micael Silva[3,4], Ēriks Kupče[5], Sundaresan Jayanthi[1,6], Adonis Lupulescu[1], Rina Rosenzweig[3], and Lucio Frydman[1*]

[1]Departments of Chemical and Biological Physics, Weizmann Institute of Science, Rehovot 7610001, Israel

[2]Department of Chemistry Education, Kyungpook National University, Daegu 41566, South Korea

[3]Departments of Chemical and Structural Biology, Weizmann Institute of Science, Rehovot 7610001, Israel

[4]Present address: Department of Pharmaceutical Sciences, University of Vienna, Josef-Holaubek-Platz 2, 1090 Vienna, Austria

[5]Latvian Academy of Sciences, Alademijas Laukums 1, Riga, Latvia.

[6]Department of Physics, Indian Institute of Space Science and Technology, Valiamala, Thiruvananthapuram 695 547, Kerala, India

*Email: lucio.frydman@weizmann.ac.il

Table of Contents

Table S1…………..…………………………………………………………………..S3

Figure S1………………………..…………………………………………………S4

Figure S2. …………..……………………………………………..……………S5

Figure S3…………..…………………………………………………...………S6

Figure S4…………..……………………………………………….…………S11

Figure S5. …………..………………………………………………………S16

Figure S6…………..…………………………………………………………S17

Figure S7..…………..…………………………………………………………S18

Figure S8. …………..………………………………………………………S19

Figure S9. …………..………………………………………………………S22

Figure S10..…………..…………………………………………..………S23

Table S2..…………..……………………………………………………...……S24

Figure S11. …………..………………………………………………………..S25

Table S3...…………..…………………………………………………………S26

Figure S12..…………..………………………………………………………..S27

Figure S13..…………..…………………………..……………………………S28

Table S1. Parameters used for conventional, Extended Hadamard, and DANTE CEST experiments.

	drkN SH3			hTRF1		
	Conv. CEST	eHT CEST	DANTE CEST	Conv. CEST	eHT CEST	DANTE CEST
NS	8	4	4	8	4	4
# points in CEST dimension	63	63	28 (SW 780 Hz) 29 (SW 810 Hz)	102	62	28 (SW 780 Hz) 29 (SW 810 Hz)
Step size (Hz)	50	50	30	30	50	30
Saturation pulse	CW	PC9 (BW 40 Hz)	DANTE	CW	*sinc450* (BW 28 Hz)	DANTE
Total exchange delay (T_{EX}, ms)	500	180x3 = 540	500	300	101x3 = 303	300
γB_1^{eff} (Hz)	30	2.2	30*	20	21	20*
^{1}H decoupling	Random dec.	Random dec.	90x240y90x	Random dec.	Random dec.	90x240y90x
Total experimental time (hr)	19.5	19.9 (H64x2)	18 (two datasets)	28.3	17.8 (H32x4)	19.4 (two datasets)

* Effective B1: $B_1^{D-CEST}\frac{\tau_\rho}{\tau'}$, with τ_ρ the width of the pulses in the DANTE train and τ' the interpulse separation in the train

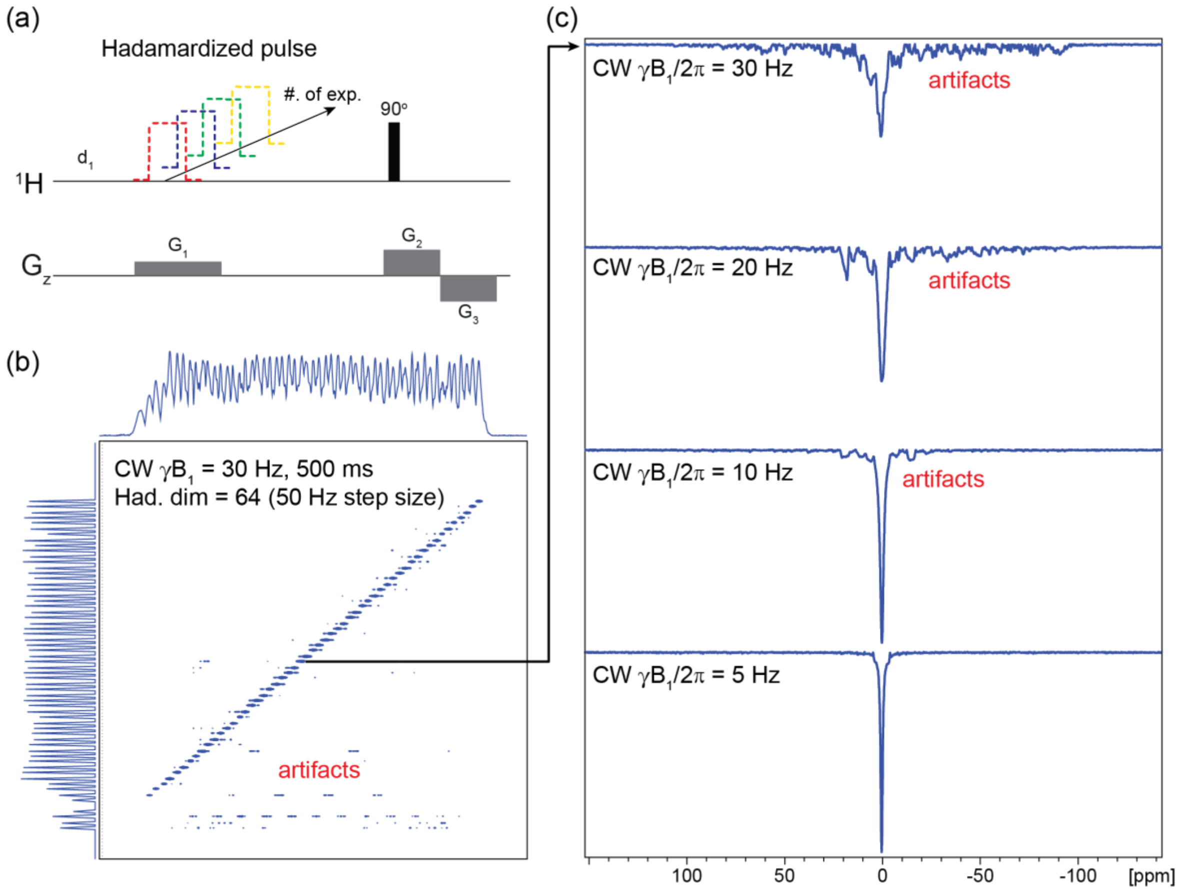

Figure S1. (a) Pulse sequence developed for checking the saturation profiles resolution that can be expected from CW-based Hadamard polychromatic pulses. A total of 64 Hadamard pulses, encoding 64 saturation frequencies spaced 50 Hz apart, were applied during the G_1 gradient, followed by a 1D (gradient echo) image acquisition. In all cases, square profiles were used as envelops for the arrays. (b) 2D "spectrum" (magnitude mode) arising after applying a Hadamard decoding process to the 64 gradient-echo images obtained upon implementing the sequence in (a). These results are displayed in a pseudo-2D format, where each image resolved by the Hadamard transform is plot horizontally, after being placed at its correct frequency bin in the vertical dimension. Peaks on the diagonal represent the frequencies encoded by the Hadamard saturation matrix, with artifacts appearing in off-diagonal positions. Most of these arise towards the edge of the sample and can be attributed to gradient non-linearities; those closer to the center of the sample, however, arise from cross-talk among the saturation pulses. (c) Spectra extracted from (b) as a function of the γB_1 fields used to compose the Hadamard pulses; artifacts decrease and eventually disappear as the γB_1 field used for each frequency decreases to 5 Hz.

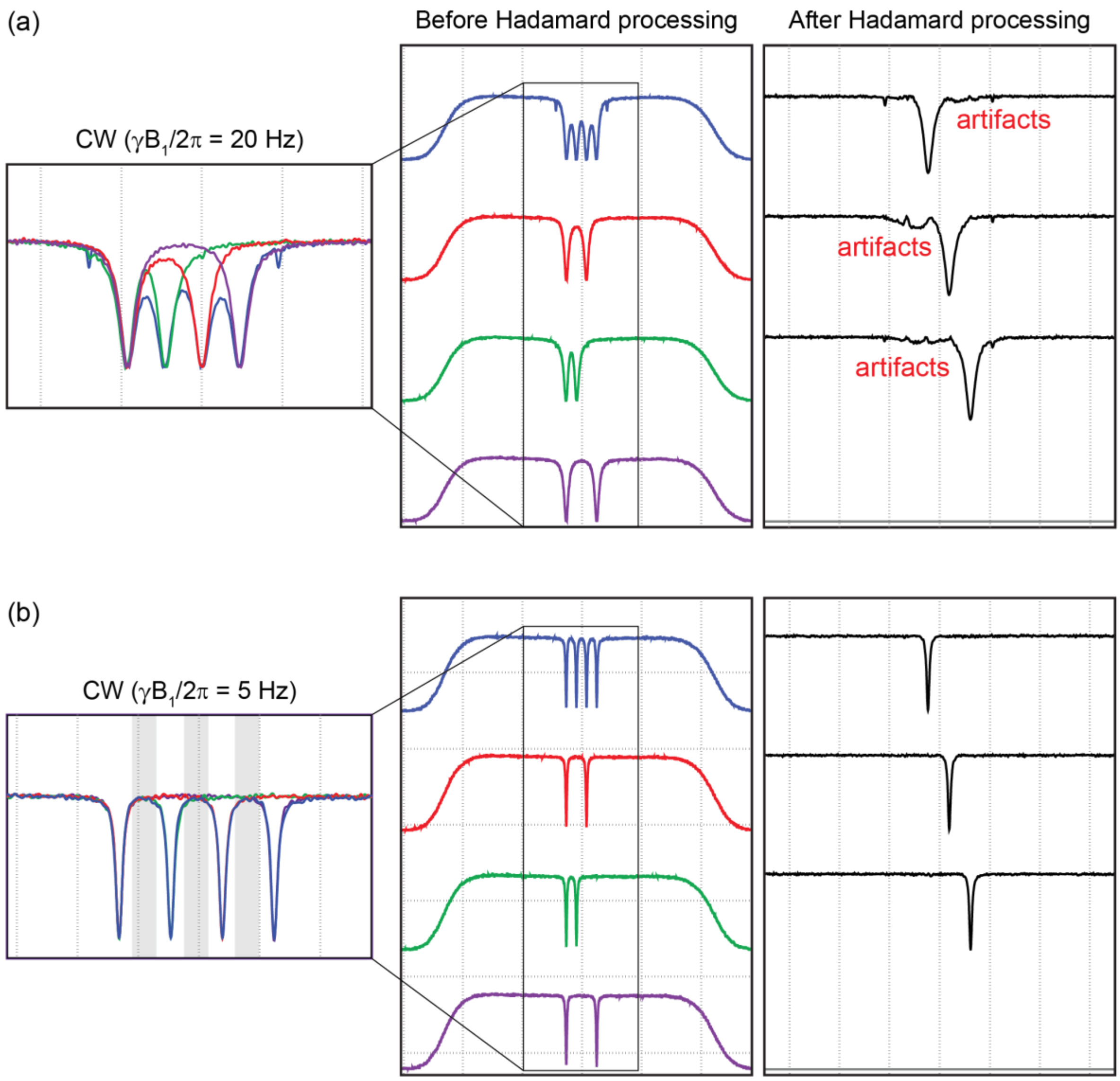

Figure S2. NMR images of a doped water sample measured using the experiment in Fig S1a and CW-based Hadamard-encoded saturation pulses with (a) $\gamma B_1/2\pi$ = 20 Hz, (b) $\gamma B_1/2\pi$ = 5 Hz. For the sake of simplicty, only 4 frequencies were selected and encoded in the Hadamard matrix. Displayed in the center column are the saturation profiles measured with each "Hadamardized" pulse, as "burned" in the gradient-imposed distribution. Illustrated on the right are the single-signal spectra unraveled at each selected frequency by the Hadamard processing. Saturation pulses in (a) cover the full width of each encoded bin but the processed "CEST spectra" show clear cross-talks between frequency bins. No artifacts are observed in (b), but the saturation pulses do not fully cover the whole span of each frequency bin due to their narrow bandwidth.

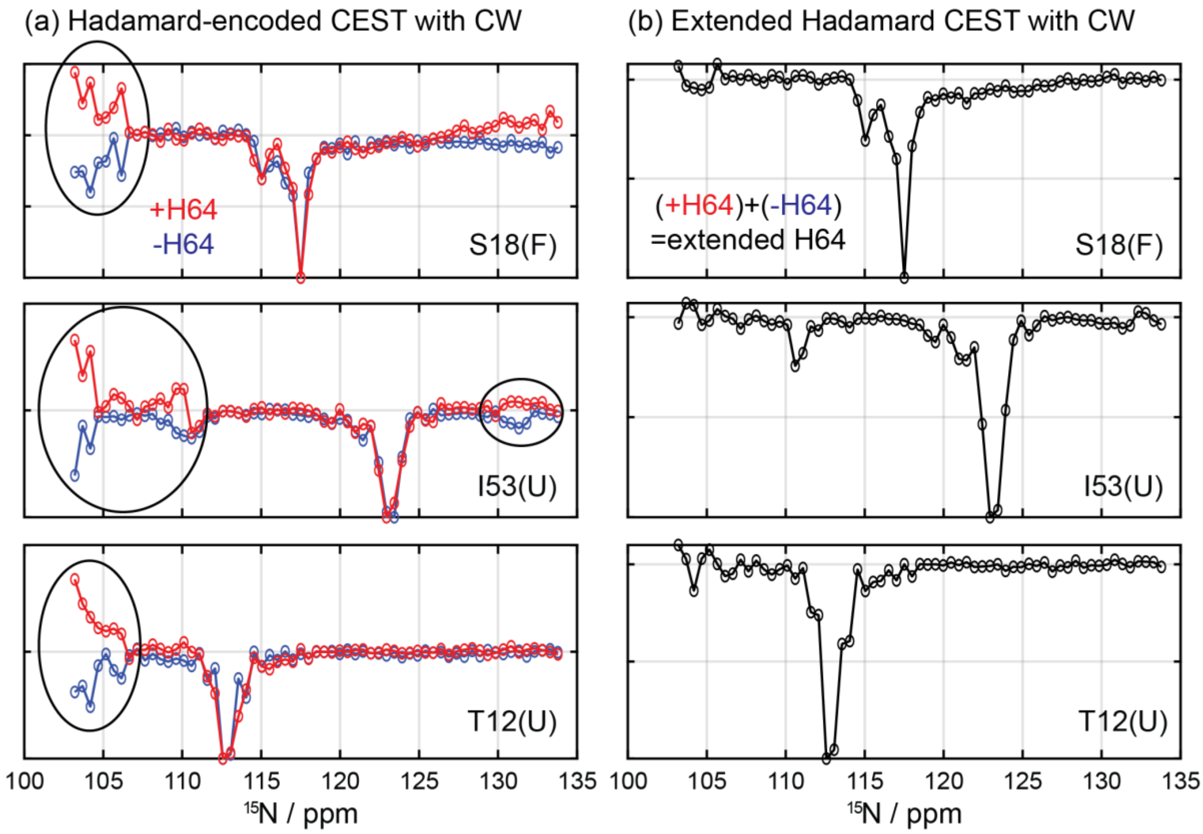

Figure S3. Idem as in Figure 4 of the main text but measured by executing the Hadamard CEST pulse sequence using CW (square-shaped) polychromatic pulses with $\gamma B_1/2\pi = 20$ Hz (same as used in Figure S2). Note that although some artifacts are cancelled out by summing the profiles measured using +H64 and -H64, the baseline is worse than that obtained with PC9-based polychromatic pulses (Figure 4) due to overlap and crosstalk among the saturation pulses.

Comparison of ^{15}N CEST profiles for 1.2 mM drkN SH3 for conventional, extended Hadamard, and D-CEST experiments

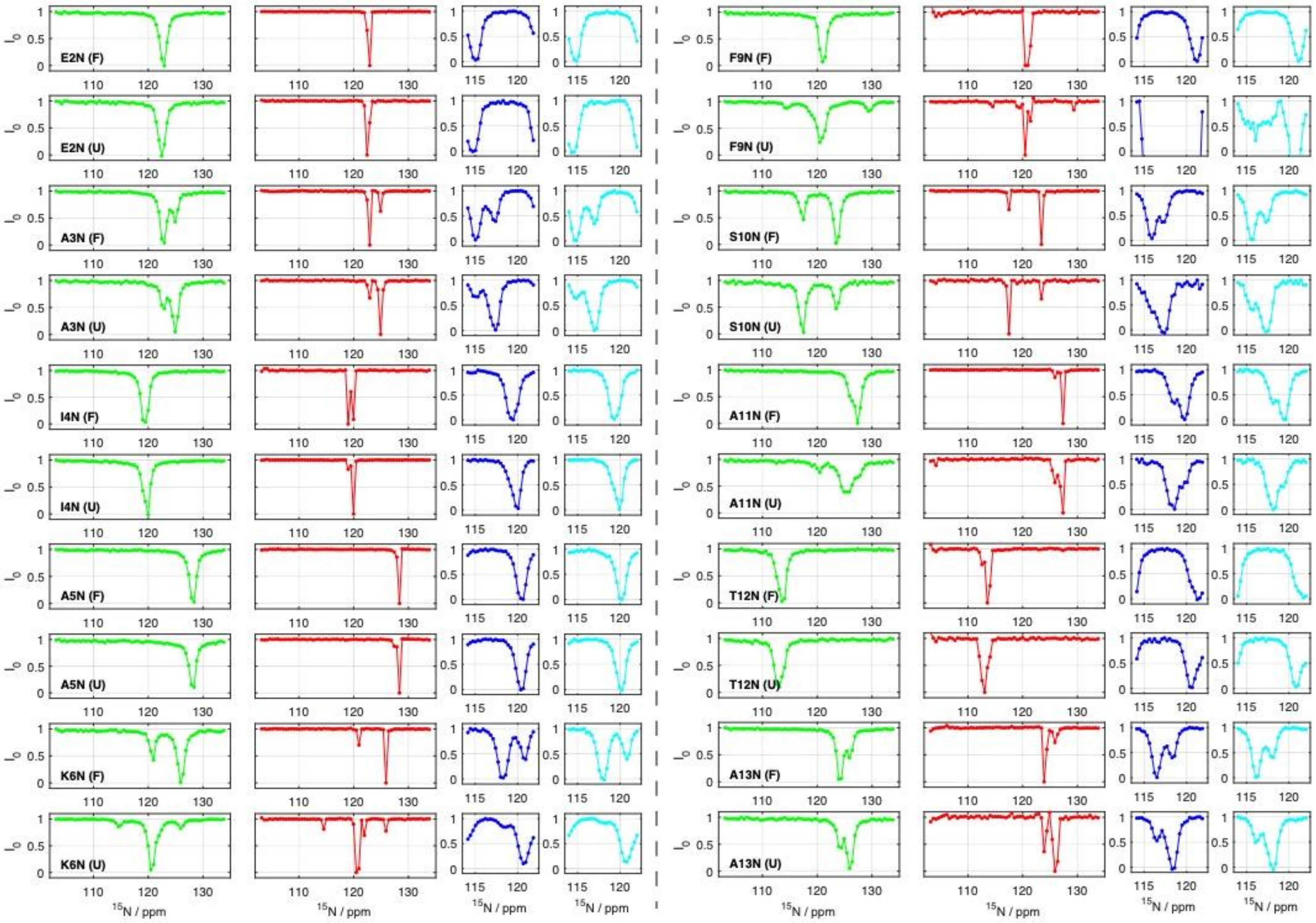

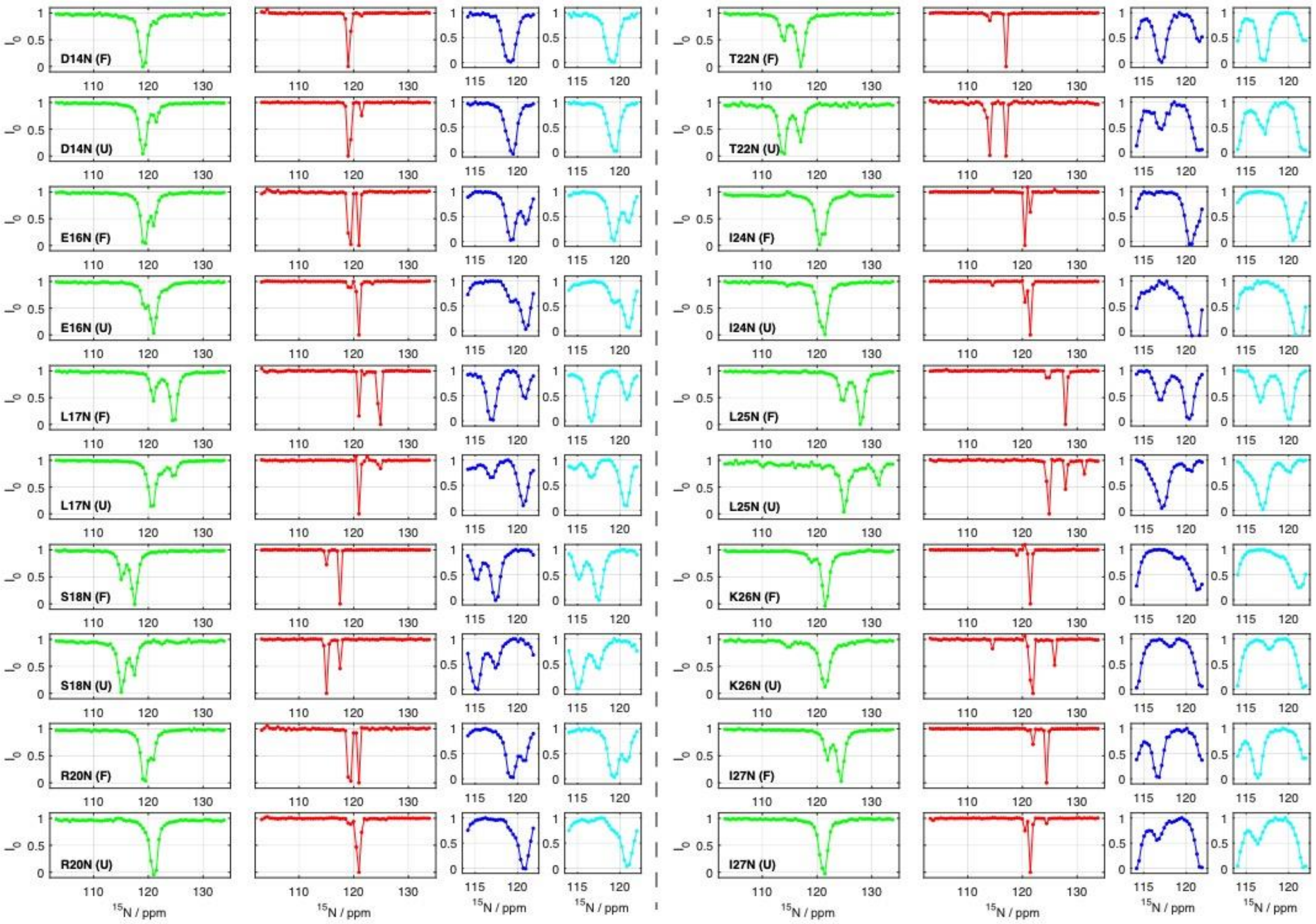
D14N (F)
D14N (U)
E16N (F)
E16N (U)
L17N (F)
L17N (U)
S18N (F)
S18N (U)
R20N (F)
R20N (U)
T22N (F)
T22N (U)
I24N (F)
I24N (U)
L25N (F)
L25N (U)
K26N (F)
K26N (U)
I27N (F)
I27N (U)
^{15}N / ppm

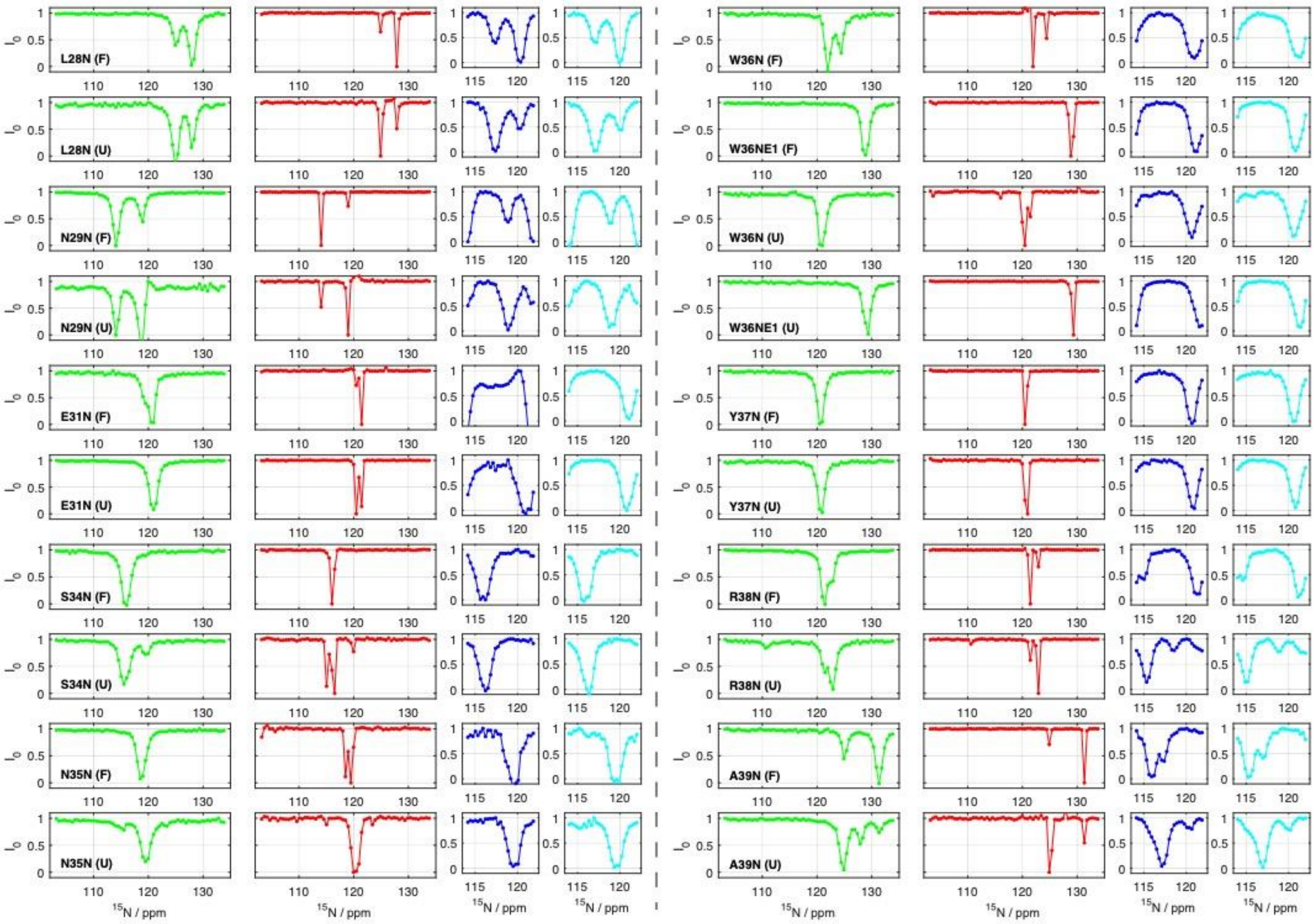
L28N (F)
L28N (U)
N29N (F)
N29N (U)
E31N (F)
E31N (U)
S34N (F)
S34N (U)
N35N (F)
N35N (U)
W36N (F)
W36NE1 (F)
W36N (U)
W36NE1 (U)
Y37N (F)
Y37N (U)
R38N (F)
R38N (U)
A39N (F)
A39N (U)
I/I0
15N / ppm

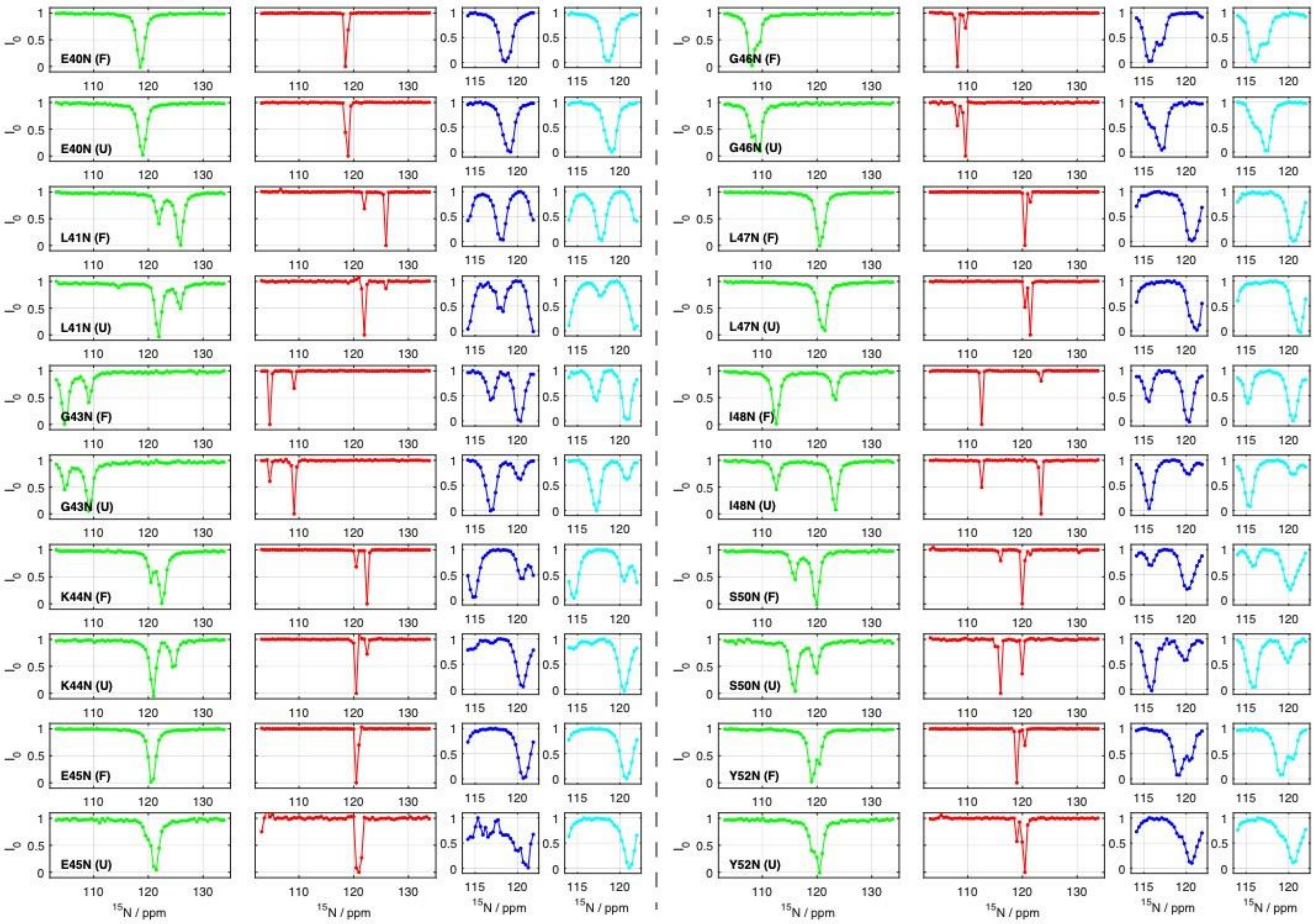

E40N (F)
E40N (U)
L41N (F)
L41N (U)
G43N (F)
G43N (U)
K44N (F)
K44N (U)
E45N (F)
E45N (U)
G46N (F)
G46N (U)
L47N (F)
L47N (U)
I48N (F)
I48N (U)
S50N (F)
S50N (U)
Y52N (F)
Y52N (U)
^{15}N / ppm

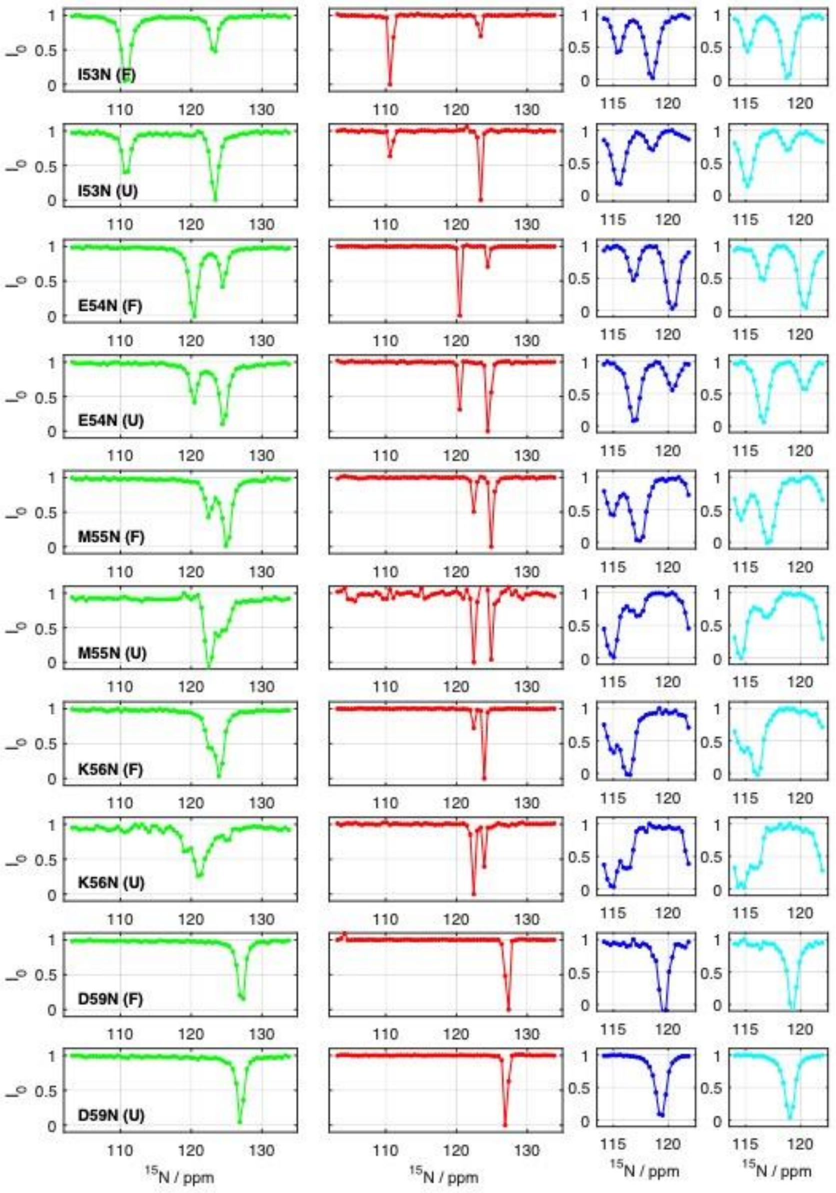

Figure S4. ^{15}N CEST profiles of 1.2 mM drkN SH3 measured at 298 K and 1.0 GHz NMR using conventional CEST (green), extended Hadamard CEST (red), and DANTE CEST (blue and cyan) with two different spectral widths (780 and 810 Hz). To compare resolution and sensitivity, all profiles are plotted with normalized intensity, setting the maximum intensity value to 1. The γB_1 offset ranges in each CEST experiment are as follows: [-1500, 1600] Hz for conventional and extended Hadamard CEST, and [-390, 390] Hz and [-405, 405] Hz for DANTE CEST. In both conventional and DANTE CEST experiments, an γB_1^{eff} field of 30 Hz with a duration of 500 ms was used. For extended Hadamard CEST, a PC9 pulse with an effective bandwidth of approximately 40 Hz and an γB_1^{eff} field of about 2.2 Hz was used.

Comparison of ^{15}N CEST profiles for 100 μM drkN SH3 for conventional, extended Hadamard, and D-CEST experiments

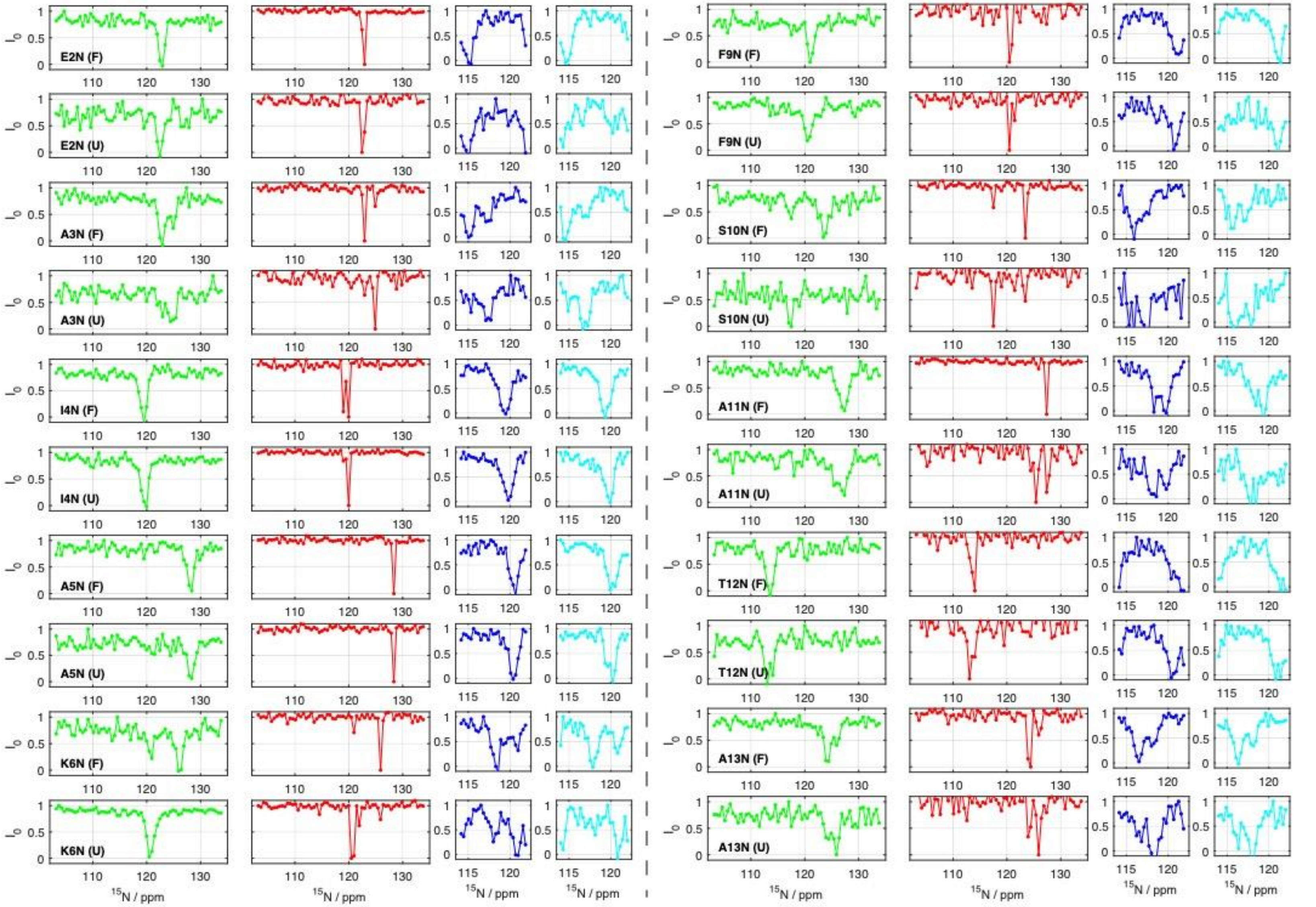

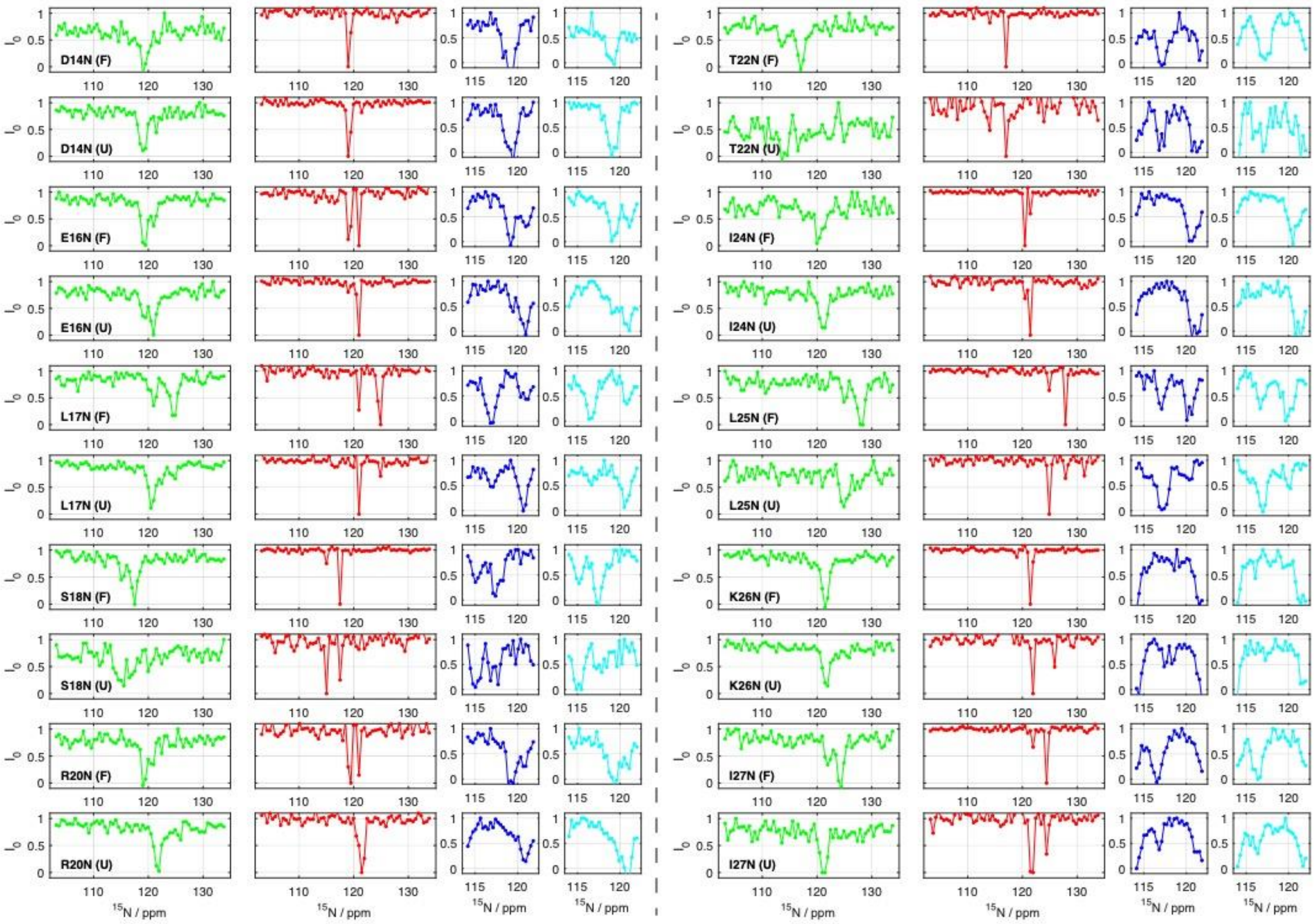

D14N (F)
D14N (U)
E16N (F)
E16N (U)
L17N (F)
L17N (U)
S18N (F)
S18N (U)
R20N (F)
R20N (U)
T22N (F)
T22N (U)
I24N (F)
I24N (U)
L25N (F)
L25N (U)
K26N (F)
K26N (U)
I27N (F)
I27N (U)
^{15}N / ppm

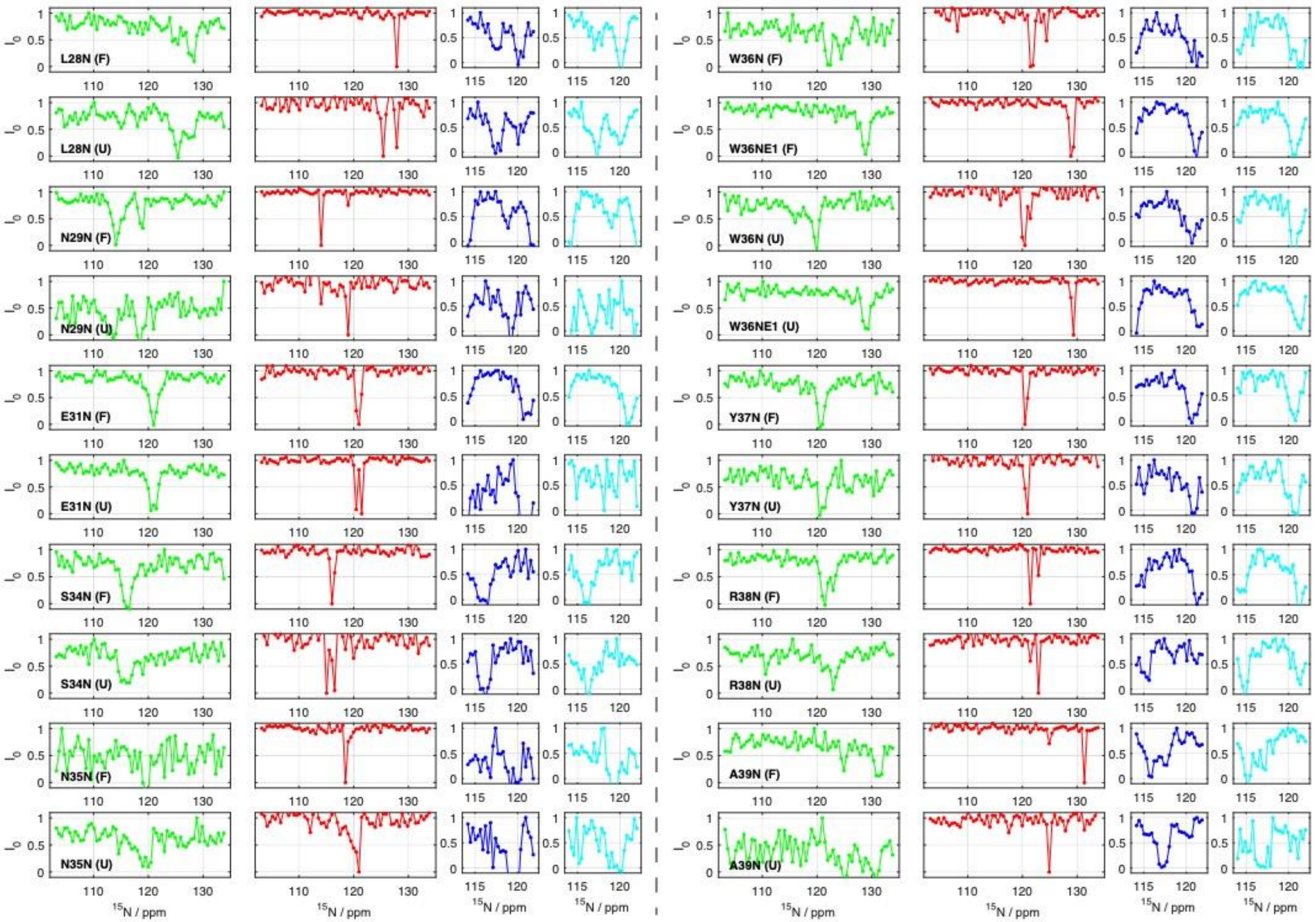

L28N (F)
L28N (U)
N29N (F)
N29N (U)
E31N (F)
E31N (U)
S34N (F)
S34N (U)
N35N (F)
N35N (U)
W36N (F)
W36NE1 (F)
W36N (U)
W36NE1 (U)
Y37N (F)
Y37N (U)
R38N (F)
R38N (U)
A39N (F)
A39N (U)
^{15}N / ppm

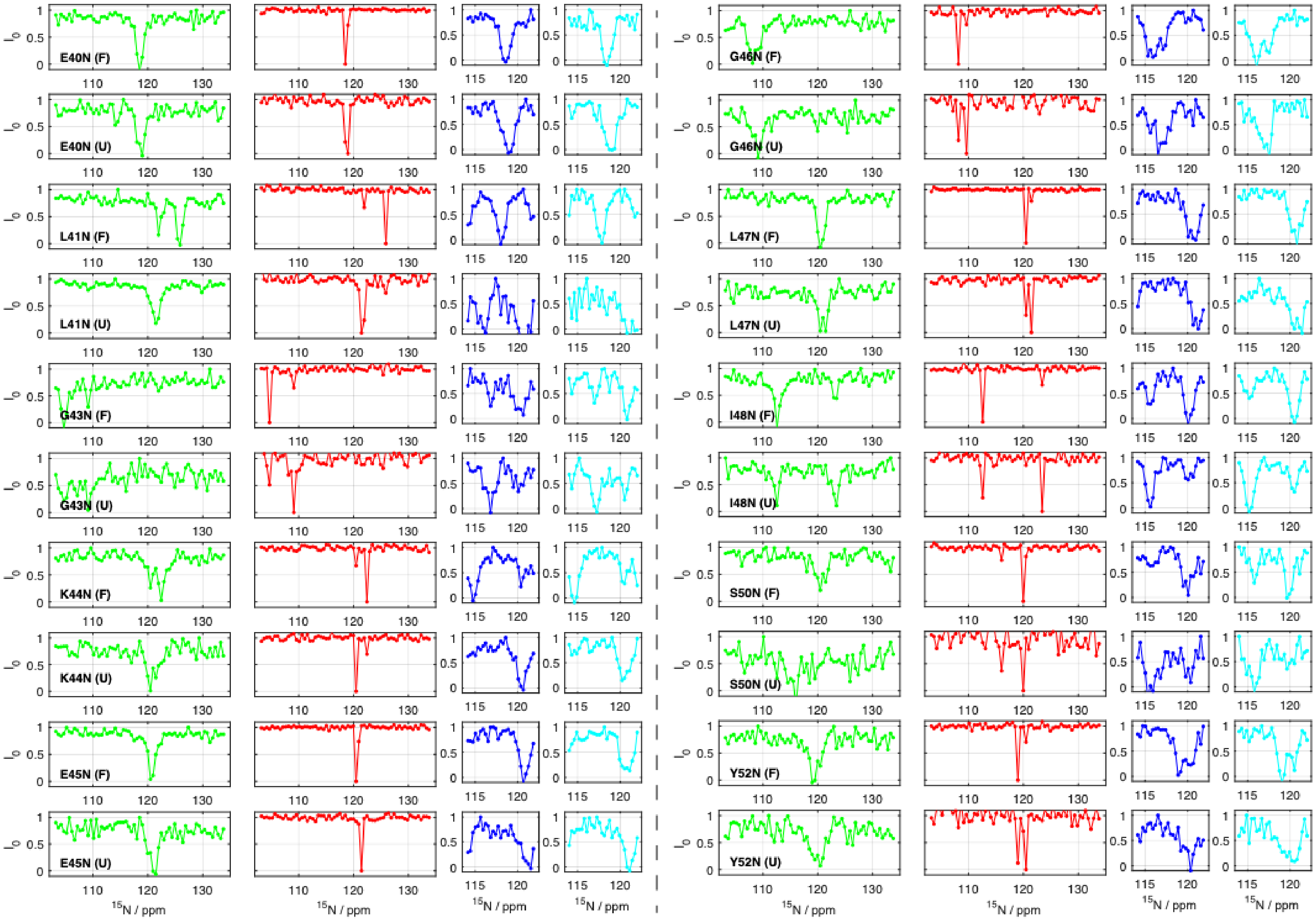

E40N (F)
E40N (U)
L41N (F)
L41N (U)
G43N (F)
G43N (U)
K44N (F)
K44N (U)
E45N (F)
E45N (U)
G46N (F)
G46N (U)
L47N (F)
L47N (U)
I48N (F)
I48N (U)
S50N (F)
S50N (U)
Y52N (F)
Y52N (U)
^{15}N / ppm

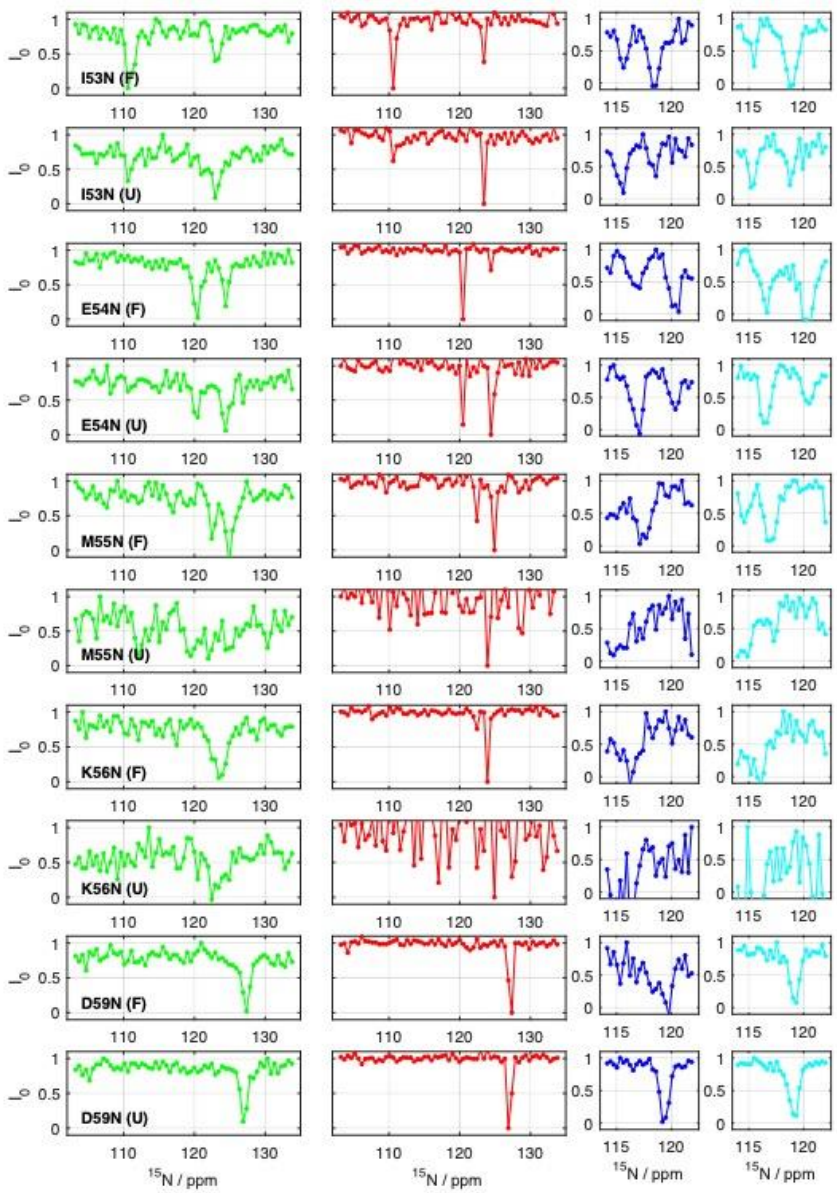

Figure S5. Idem as Figure S4, but for 0.1 mM drkN SH3.

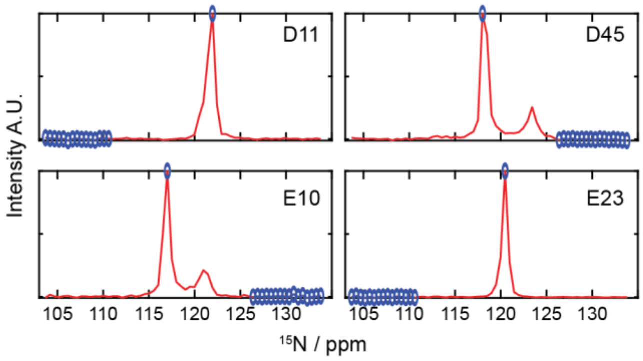

Figure S6. Illustration of SNR calculations for the CEST profiles. Profiles from both conventional and extended Hadamard CEST were plotted in absolute intensity, *I*, with the intensity dip of the main state set to show the maximum value. In the conventional case, the profile is plotted as *-I,* with the baseline adjusted to zero. The SNR was then calculated as maximum peak intensity / σ_{noise} (marked as blue circles). To account for differences in acquisition time, SNRT (SNR per unit time) factors were defined as: $SNRT = SNR/\sqrt{total\ experimental\ time}$

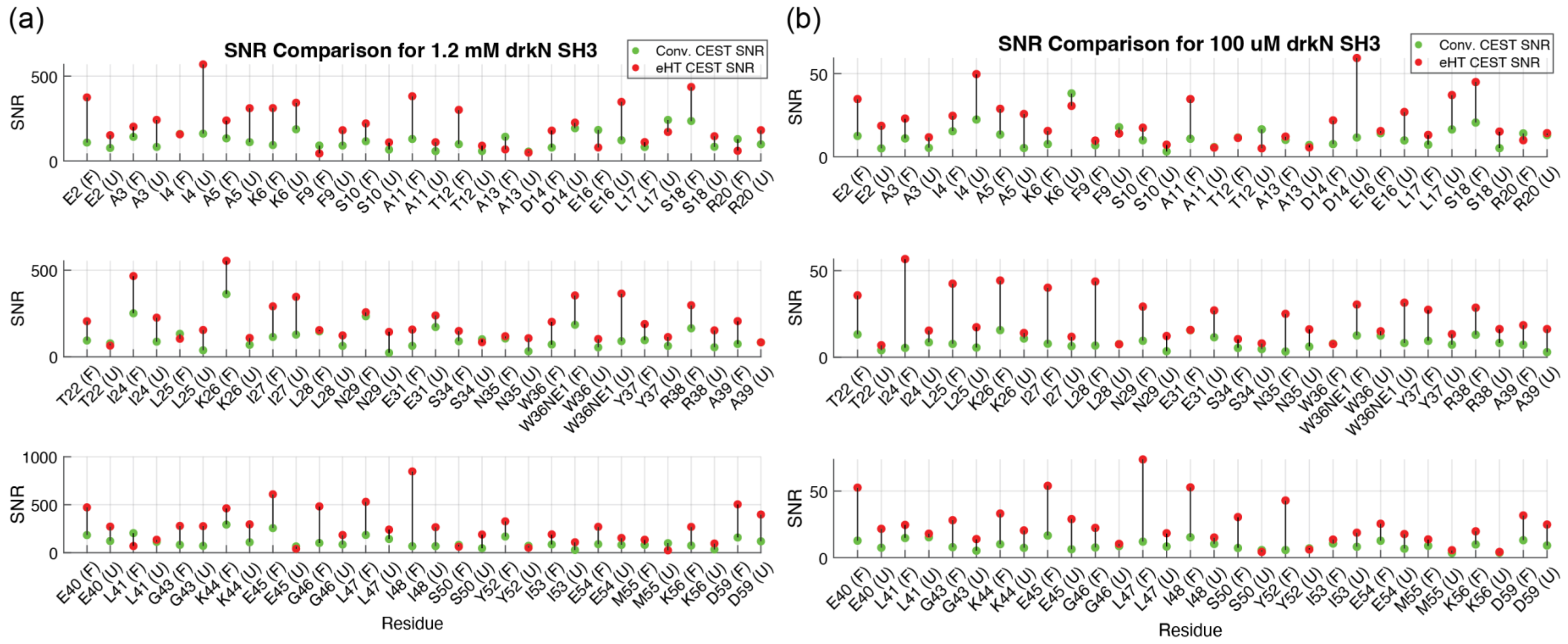

Figure S7. Comparison of SNR from CEST profiles between conventional CEST (green) and extended Hadamard CEST (red) for residues of (a) 1.2 mM drkN SH3 and (b) 0.1 mM drkN SH3. Since the acquisition times for both experiments are nearly the same, the averaged SNR and SNRT values are almost identical: (a) average SNR (SNRT) = 2.3 and (b) = 2.6.

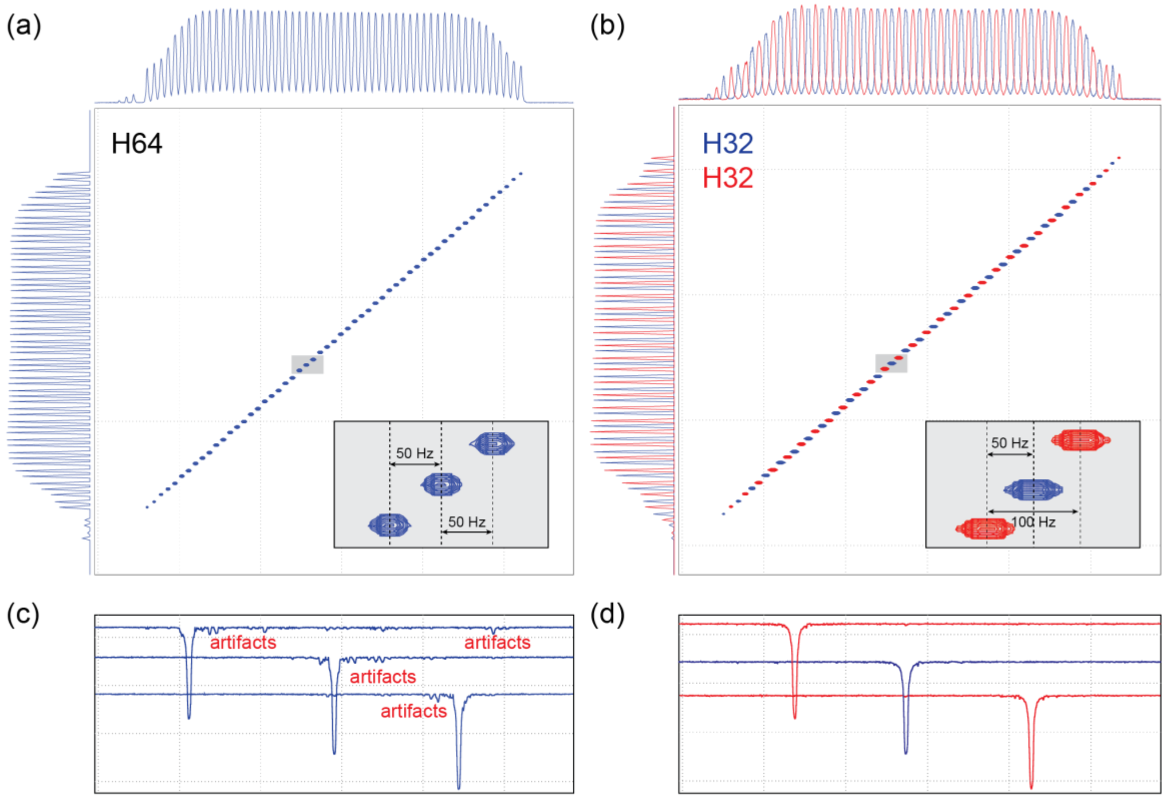

Figure S8. Saturation profile of the *sinc450*-shaped Hadamard pulse with a bandwidth of 28 Hz, corresponding to an γB_1^{eff} of 21 Hz. In (a), a total of 64 frequencies are encoded in H64, with the frequencies separated by 50 Hz. In (b), the same 64 frequencies are encoded across two H32 matrices, with frequencies separated by 100 Hz. Note that the artifacts arising from overlapping neighbouring pulses in (a) disappear in (b).

Comparison of ^{15}N CEST profiles for 1.2 mM hTRF1 for conventional, extended Hadamard, and D-CEST experiments

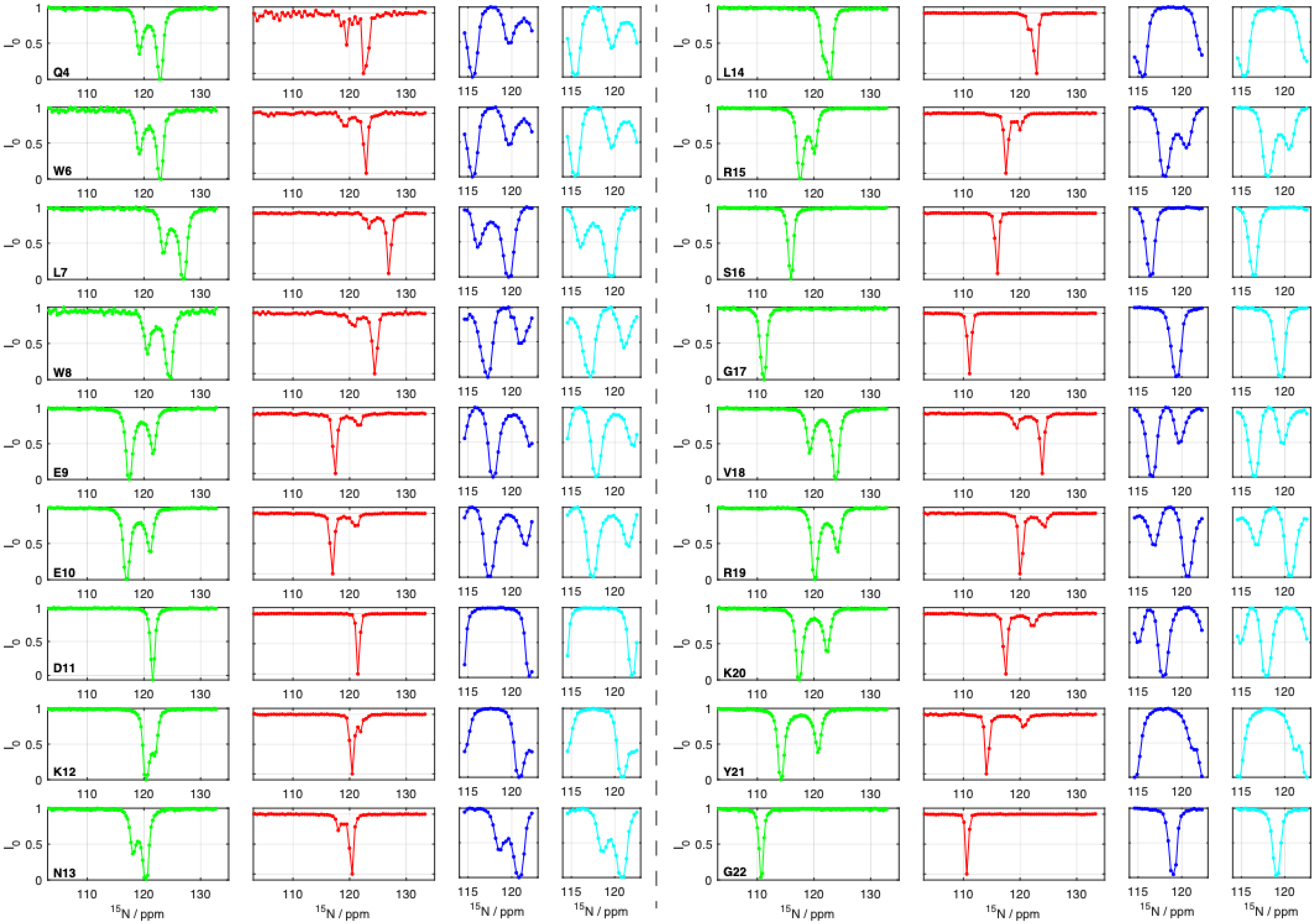

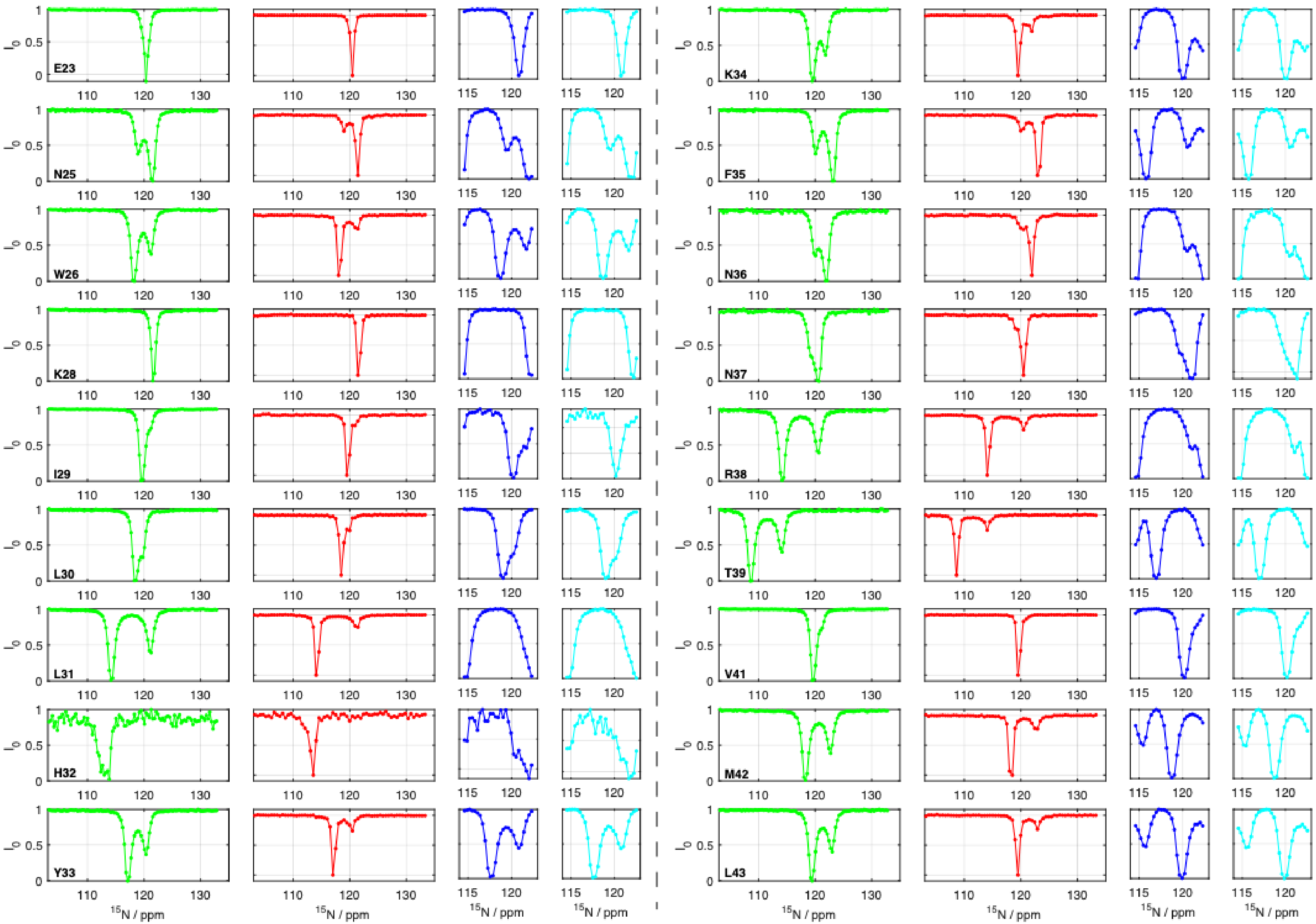
E23
N25
W26
K28
I29
L30
L31
H32
Y33
K34
F35
N36
N37
R38
T39
V41
M42
L43
^{15}N / ppm

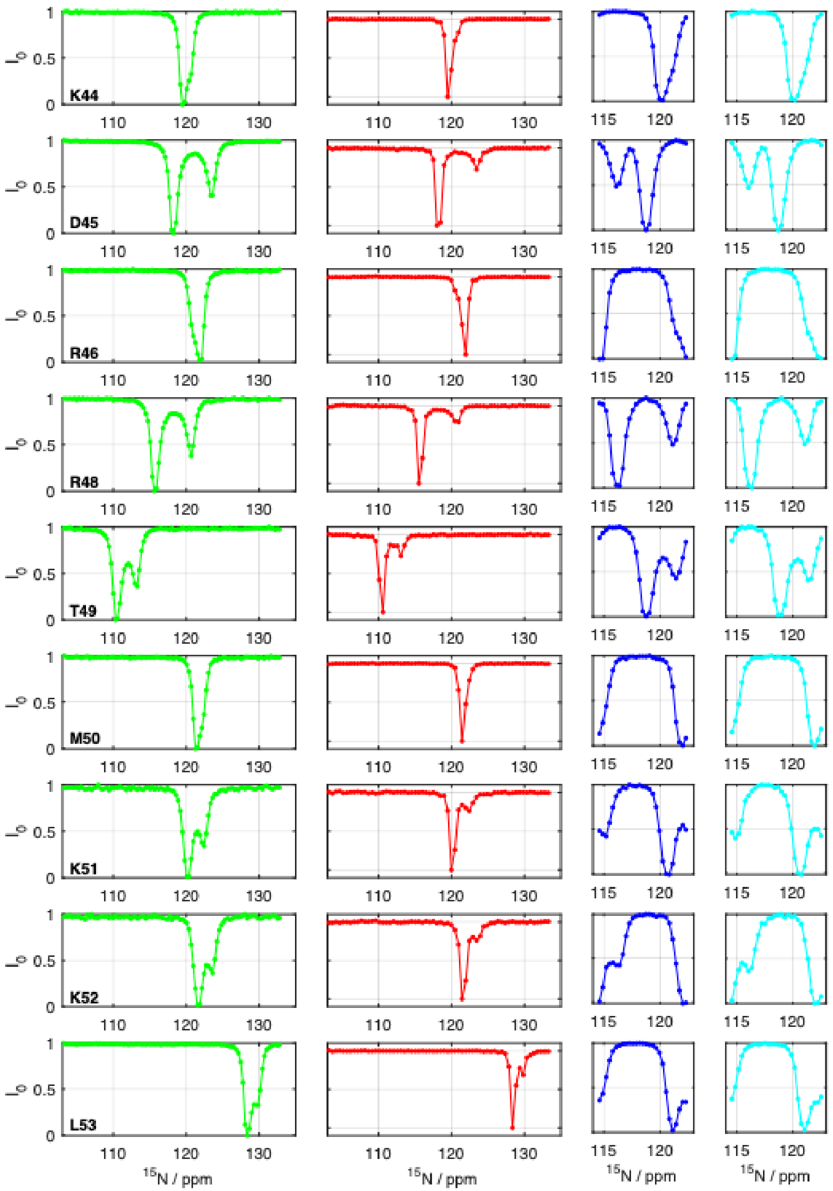

Figure S9. ^{15}N CEST profiles of 1.2 mM hTRF1 measured at 309.5 K and 1.0 GHz NMR using conventional CEST (green), extended Hadamard CEST (red), and DANTE CEST with two different spectral widths (790 and 800 Hz, blue and cyan respectively). To compare resolution and sensitivity, all profiles are plotted with normalized intensity, setting the maximum intensity value to 1. The γB_1^{eff} offset ranges in each CEST experiment were as follows: [-1500, 1500] Hz for conventional and extended Hadamard CEST, and [-395, 395] Hz and [-400, 400] Hz for DANTE CEST. In both experiments, a γB_1^{eff} field of 20 Hz with a T_{EX} of 300 ms was applied. For the extended Hadamard CEST, 32 *sinc450* pulse elements with individual effective bandwidths of approximately 28 Hz and γB_1^{eff} fields of about 21 Hz was used in 32-binned combinations.

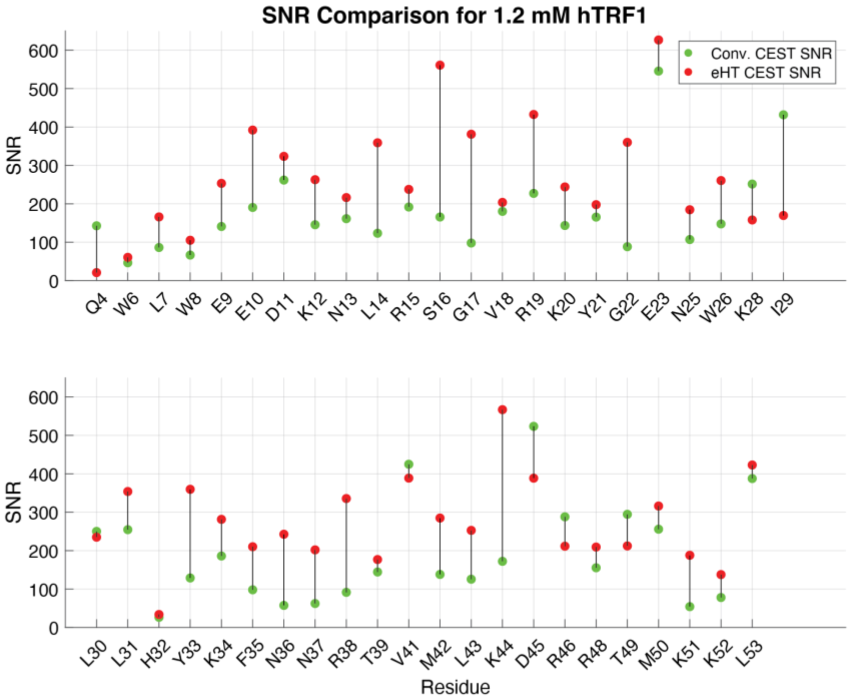

Figure S10. Idem as Figure S7, but for 1.2 mM hTRF1. Averaged SNR and SNRT are 1.8 and 2.3, respectively.

Table S2. ^{15}N chemical shift difference (Δω) between the F and U states of the drkN SH3 at 298 K measured at 1 GHz as extracted from fits of both conventional and DANTE CEST profiles by ChemEx using a two-state exchange model.

	**Conventional**		**DANTE**	
	**Δω (ppm)**	**error**	**Δω (ppm)**	**error**
**E2**	-0.42	0.03	-0.59	0.03
**A3**	2.23	0.02	2.26	0.01
**I4**	0.77	0.02	0.75	0.02
**A5**	-0.05	0.02	-0.05	0.04
**K6**	-5.2	0.02	-5.19	0.02
**D8**	2.43	0.02	2.43	0.03
**F9**	-0.71	0.03	-0.55	0.03
**S10**	-6.27	0.01	-6.26	0.01
**A11**	-1.32	0.02	-1.4	0.02
**T12**	-0.91	0.04	-0.93	0.04
**A13**	1.93	0.01	1.94	0.01
**D14**	0.33	0.07	0.33	0.08
**D15**	5.82	0.01	5.84	0.01
**E16**	1.84	0.01	1.86	0.01
**L17**	-3.71	0.02	-3.68	0.03
**S18**	-2.38	0.01	-2.37	0.01
**F19**	6.91	0.01	6.92	0.02
**R20**	1.6	0.01	1.62	0.02
**K21**	0.32	0.03	0.05	0.05
**T22**	-3.26	0.01	-3.3	0.02
**L25**	-3.4	0.01	-3.43	0.01
**I27**	-2.51	0.01	-2.62	0.03
**L28**	-3.02	0.02	-3.06	0.02

	**Conventional**		**DANTE**	
	**Δω (ppm)**	**error**	**Δω (ppm)**	**error**
**N29**	4.72	0.01	4.7	0.03
**M30**	-2.93	0.03	-2.94	0.03
**S34**	0.36	0.06	0.63	0.04
**W36**	-1.08	0.05	-0.78	0.07
**Y37**	0.12	0.06	0.02	0.05
**R38**	1.48	0.02	1.45	0.03
**A39**	-6.32	0.01	-6.3	0.02
**E40**	0.47	0.03	0.57	0.04
**L41**	-3.9	0.01	-3.91	0.01
**D42**	-9.07	0.02	-9	0.05
**G43**	4.35	0.02	4.34	0.02
**K44**	-2.08	0.01	-2.1	0.01
**E45**	-0.26	0.05	0.67	0.04
**G46**	1.45	0.01	1.43	0.02
**L47**	0.81	0.01	0.59	0.06
**I48**	10.66	0.02	10.64	0.02
**S50**	-4.51	0	-4.44	0.05
**I53**	12.42	0.01	12.37	0.01
**E54**	4.17	0.01	4.19	0.03
**M55**	-2.55	0.02	-2.52	0.02
**K56**	-1.48	0.02	-1.46	0.03
**D59**	-0.43	0.02	-0.41	0.03

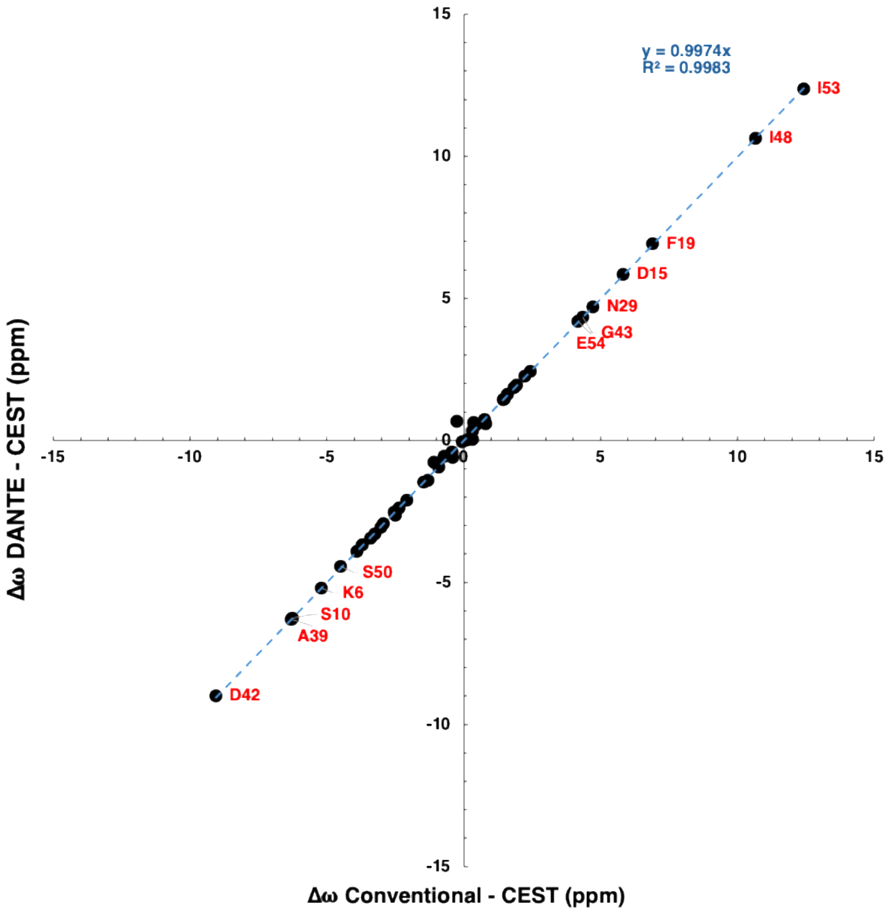

Figure S11. Correlation plot between Δω of drkN SH3 from conventional and DANTE-CEST experiments as extracted from fits by ChemEX. The values are in good agreement as demonstrated by the linear trendline with $R^2 = 0.998$.

Table S3. ^{15}N chemical shift difference (Δω) between the F and U states of the hTRF1 at 309.5 K measured at 1 GHz as extracted from fits of conventional and DANTE CEST profiles by ChemEX using a two-state exchange model.

	**Conventional**		**DANTE**	
	**Δω (ppm)**	**error**	**Δω (ppm)**	**error**
**W8**	-4.08	0.03	-4.08	0.03
**E9**	4.33	0.01	4.3	0.01
**E10**	4.25	0.01	4.25	0.01
**D11**	-0.36	0.01	-0.41	0.01
**K12**	1.74	0.01	1.7	0.01
**N13**	-2.4	0.01	-2.41	0.01
**L14**	-1.38	0.01	-1.35	0.01
**R15**	2.64	0.01	2.64	0.01
**S16**	-0.48	0.01	-0.53	0.01
**G17**	-0.63	0.01	-0.65	0.01
**R19**	4.07	0.01	4.07	0.01
**G22**	-0.34	0.01	-0.41	0.01
**E23**	-0.14	0.03	-0.19	0.03
**N25**	-2.63	0.01	-2.64	0.02
**W26**	3.09	0.01	3.09	0.01
**L30**	1.48	0.01	1.46	0.01
**L31**	6.89	0.02	6.97	0.02
**Y33**	3.29	0.01	3.29	0.01
**K34**	2.44	0.01	2.42	0.01
**F35**	-3.17	0.01	-3.17	0.02
**N36**	-2.2	0.02	-2.21	0.03
**N37**	-1.56	0.01	-1.54	0.03
**V41**	0.79	0.02	0.77	0.04
**M42**	4.49	0.01	4.48	0.01
**L43**	4.49	0.01	4.48	0.01
**K44**	1.18	0.01	1.14	0.01
**D45**	5.27	0.00	5.26	0.01
**R46**	-1.26	0.01	-1.28	0.01

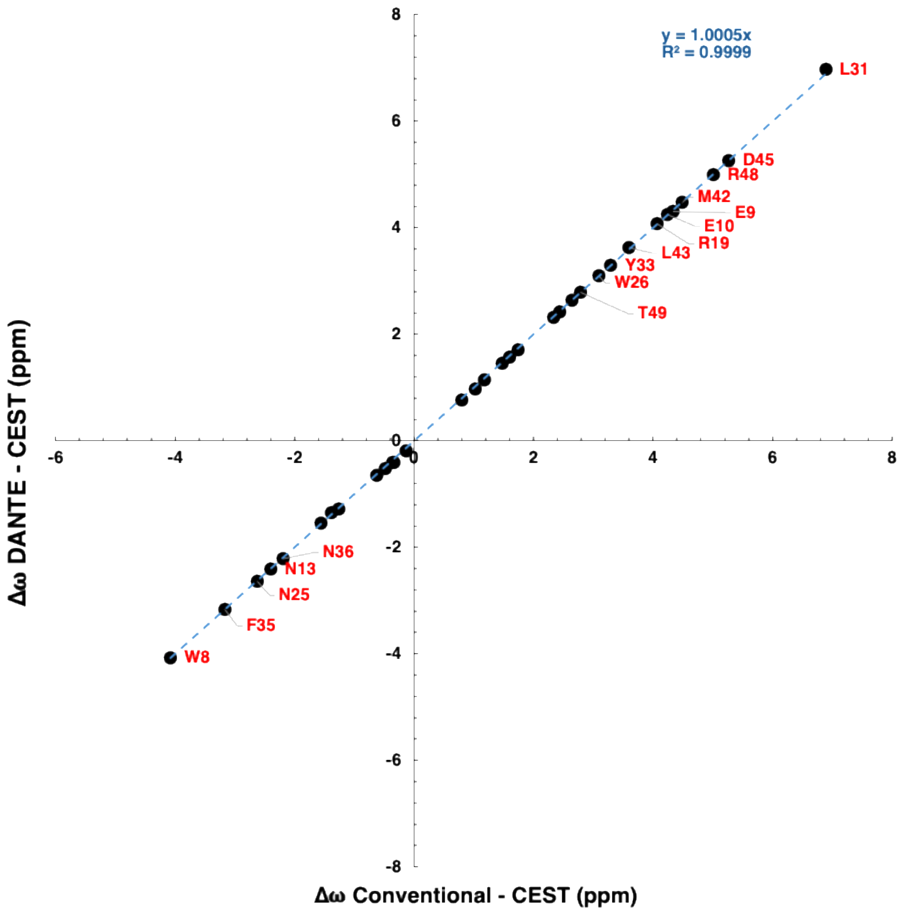

Figure S12. Correlation plot between Δω of hTRF1 from conventional and DANTE-CEST experiments as extracted from fits by ChemEx. The values are in good agreement as demonstrated by the linear trendline with $R^2 = 0.999$.

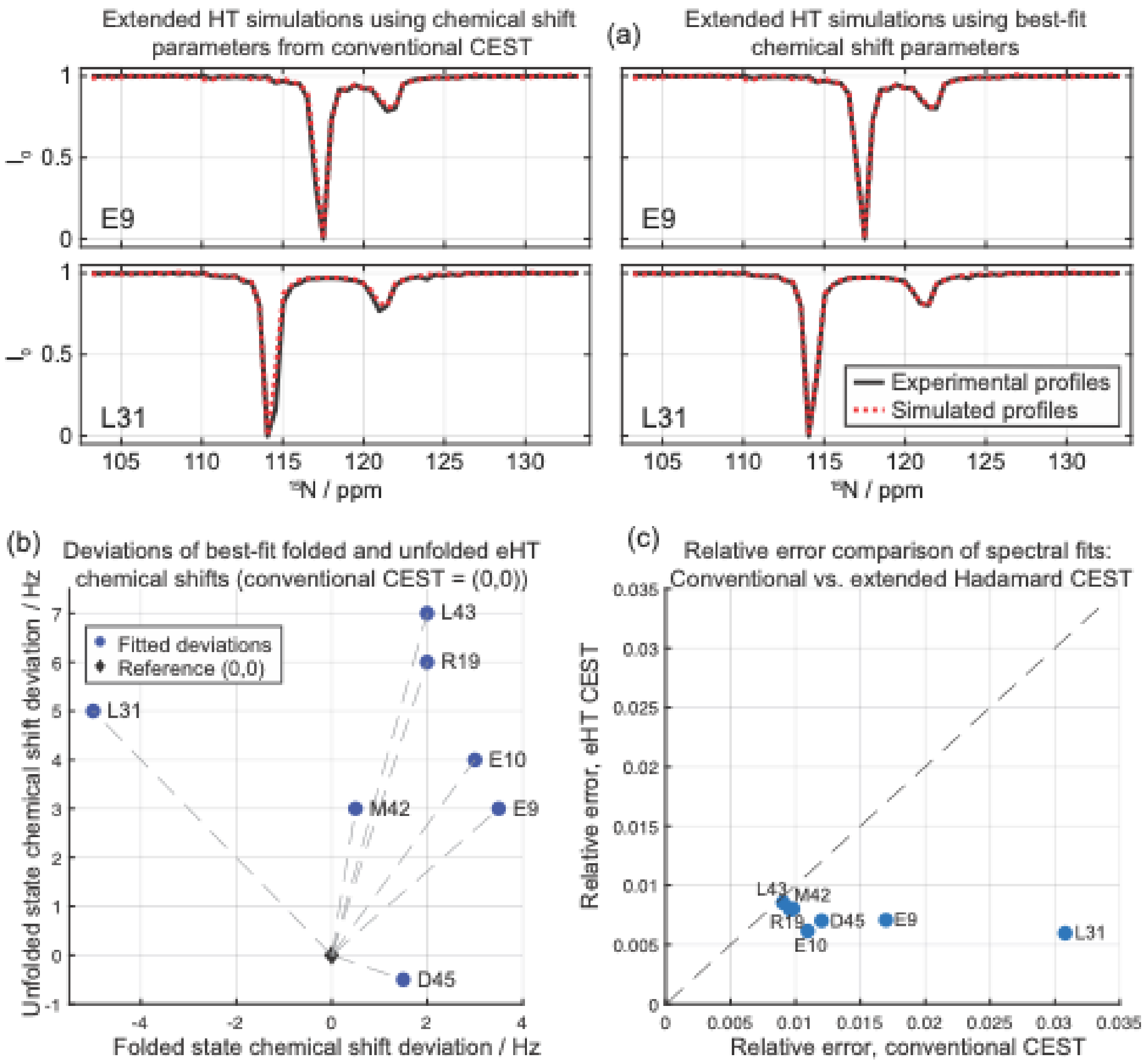

Figure S13. Idem as Figure 7 in the main text, but for the hTRF1 protein. Experiments and simulations employed *sinc450* pulses with $(\gamma B_1^{max})/2\pi = 21$ Hz and 101 ms duration. The frequency changes in Hz (ppm) for folded and unfolded conformations are: E9{3.5 (0.035), 3 (0.03)}, L13{-5 (-0.05), 5 (0.05)}. (b) Chemical shifts deviations imparted on the folded and unfolded chemical shift values of selected residues by conventional CEST, in order to obtain a best match with the Hadamard-encoded CEST experiments as in Figure 7b. (c) Correlation plot comparing the fitting errors of conventional and extended Hadamard CEST experiments, showing consistently reduced errors for the HT CEST.
